# Epileptic Seizures Detection Using Deep Learning Techniques: A Review

**DOI:** 10.3390/ijerph18115780

**Published:** 2021-05-27

**Authors:** Afshin Shoeibi, Marjane Khodatars, Navid Ghassemi, Mahboobeh Jafari, Parisa Moridian, Roohallah Alizadehsani, Maryam Panahiazar, Fahime Khozeimeh, Assef Zare, Hossein Hosseini-Nejad, Abbas Khosravi, Amir F. Atiya, Diba Aminshahidi, Sadiq Hussain, Modjtaba Rouhani, Saeid Nahavandi, Udyavara Rajendra Acharya

**Affiliations:** 1 Faculty of Electrical Engineering, Biomedical Data Acquisition Lab (BDAL), K. N. Toosi University of Technology, Tehran 1631714191, Iran; navidghassemi@mail.um.ac.ir; 2 Computer Engineering Department, Ferdowsi University of Mashhad, Mashhad 9177948974, Iran; d.aminshahidi@mail.um.ac.ir (D.A.); rouhani@um.ac.ir (M.R.); 3 Mashhad Branch, Islamic Azad University, Mashhad 91735413, Iran; khodatars1marjane@gmail.com; 4 Electrical and Computer Engineering Faculty, Semnan University, Semnan 3513119111, Iran; mahbube.jafari@yahoo.com; 5 Faculty of Engineering, Science and Research Branch, Islamic Azad University, Tehran 1477893855, Iran; parisamoridian@yahoo.com; 6 Institute for Intelligent Systems Research and Innovation (IISRI), Deakin University, Waurn Ponds, VIC 3217, Australia; ralizadehsani@deakin.edu.au (R.A.); khozeimeh@mums.ac.ir (F.K.); abbas.khosravi@deakin.edu.au (A.K.); Saeid.nahavandi@deakin.edu.au (S.N.); 7 Institute for Computational Health Sciences, School of Medicine, University of California, San Francisco, CA 94143, USA; Maryam.Panahiazar@ucsf.edu; 8 Faculty of Electrical Engineering, Gonabad Branch, Islamic Azad University, Gonabad 6518115743, Iran; assefzare@gmail.com; 9 Faculty of Electrical and Computer Engineering, K. N. Toosi University of Technology, Tehran 1631714191, Iran; hosseini_nezhad@eetd.kntu.ac.ir; 10 Department of Computer Engineering, Faculty of Engineering, Cairo University, Cairo 12613, Egypt; atiya@cario.edu; 11 System Administrator at Dibrugarh University, Assam 786004, India; sadiq@dibru.ac.in; 12 Department of Biomedical Engineering, School of Science and Technology, Singapore University of Social Sciences, Singapore 599494, Singapore; aru@np.edu.sg; 13 Department of Electronics and Computer Engineering, Ngee Ann Polytechnic, Singapore 599489, Singapore; 14 Department of Bioinformatics and Medical Engineering, Taichung City 41354, Taiwan

**Keywords:** epileptic seizures, diagnosis, EEG, MRI, feature extraction, classification, deep learning

## Abstract

A variety of screening approaches have been proposed to diagnose epileptic seizures, using electroencephalography (EEG) and magnetic resonance imaging (MRI) modalities. Artificial intelligence encompasses a variety of areas, and one of its branches is deep learning (DL). Before the rise of DL, conventional machine learning algorithms involving feature extraction were performed. This limited their performance to the ability of those handcrafting the features. However, in DL, the extraction of features and classification are entirely automated. The advent of these techniques in many areas of medicine, such as in the diagnosis of epileptic seizures, has made significant advances. In this study, a comprehensive overview of works focused on automated epileptic seizure detection using DL techniques and neuroimaging modalities is presented. Various methods proposed to diagnose epileptic seizures automatically using EEG and MRI modalities are described. In addition, rehabilitation systems developed for epileptic seizures using DL have been analyzed, and a summary is provided. The rehabilitation tools include cloud computing techniques and hardware required for implementation of DL algorithms. The important challenges in accurate detection of automated epileptic seizures using DL with EEG and MRI modalities are discussed. The advantages and limitations in employing DL-based techniques for epileptic seizures diagnosis are presented. Finally, the most promising DL models proposed and possible future works on automated epileptic seizure detection are delineated.

## 1. Introduction

Epilepsy is a noncommunicable disease and one of the most common neurological disorders of humans, usually associated with sudden attacks [1]. Sudden attacks of seizures are a swift and early abnormality in the electrical activity of the brain that disrupts the part or whole body [2]. Various kinds of epileptic seizures are affecting around 60 million people worldwide [3]. These attacks occasionally provoke cognitive disorders that can cause severe physical injury to the patient. Moreover, people with epileptic seizures sometimes suffer emotional distress due to embarrassment and lack of appropriate social status. Hence, early detection of epileptic seizures can help the patients and improve their quality of life.

Screening techniques in the diagnosis of epileptic seizures comprise two important categories of functional and structural neuroimaging modalities [4,5,6,7,8,9]. The functional neuroimaging modality provides important information about brain function during epileptic seizure occurrence for physicians and neurologists [4,5,6,7,8,9]. The structural neuroimaging modalities provide physicians with substantial information about the brain structure of individuals with epileptic seizures [4,5,6,7,8,9]. The most important functional neuroimaging techniques are EEG [5], magnetoencephalography (MEG) [6], positron emission tomography (PET) [7], single-photon emission computed tomography (SPECT) [7,10], functional MRI (fMRI) [4,11], electrocorticography (ECoG) [12], and functional near-infrared spectroscopy (fNIRS) [13]. In contrast, structural MRI (sMRI) and diffusion tensor imaging (DTI) are among the most significant structural neuroimaging techniques [4,14]. In the diagnosis of epileptic seizures, functional neuroimaging modalities are more commonly applied than structural modalities [4,5,6,7,8,9]. Research on the diagnosis of epileptic seizures has indicated that EEG modalities are the most popular among physicians. 

The EEG signals are widely preferred as they are economical, portable, and show clear rhythms in the frequency domain [8,9]. The EEG provides the voltage variations produced by the ionic current of neurons in the brain, which indicate the brain’s bioelectric activity [15]. They need to be recorded for a long period of time to detect epileptic seizures. In addition, these signals are recorded in multiple channels, making the analysis complex. The EEG signals are also prone to artifacts generated by main power supply, electrode movement, and muscle tremor [16]. This will pose challenges to the physicians to diagnose epileptic seizures using noisy EEG signals. To resolve these difficulties, much research is being carried out to diagnose and predict epileptic seizures based on EEG modalities and other techniques such as MRI coupled with AI techniques [17,18]. AI techniques in the field of epileptic seizures diagnosis have employed conventional machine learning and DL methods [19,20,21,22].

Many machine learning algorithms have been developed using statistical, time, frequency, time-frequency domain and nonlinear parameters to detect epileptic seizures [23,24]. In conventional machine learning techniques, the selection of features and classifiers is done by trial-and-error method [25,26]. One needs to have sound knowledge of signal processing and data mining techniques to develop an accurate model. Such models perform well for limited data. Nowadays, with the increase in the availability of data, machine learning techniques may not perform very well. Hence, the DL techniques, which are the state-of-art methods, have been employed [27,28]. DL models, unlike conventional machine learning techniques, require huge data in the training phase [29]. This is because these models have a large number of feature spaces, and in case of lack of data, they face the problem of overfitting [29].

**Figure 1:**
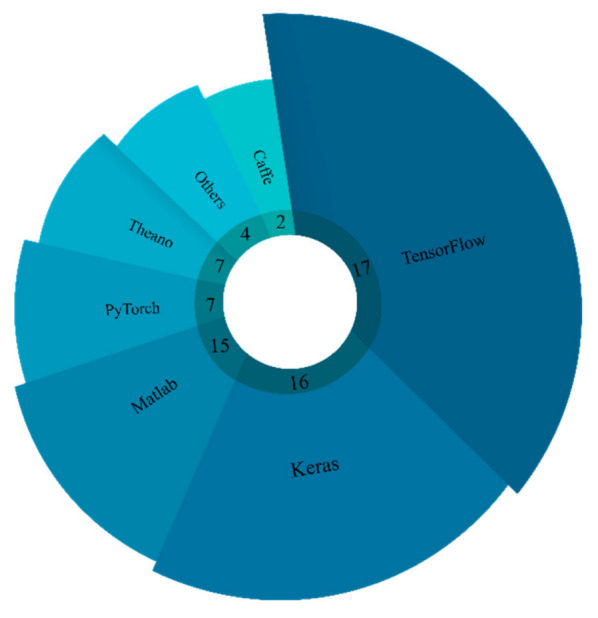
Number of times each DL tool was used for automated detection of epileptic seizure by various studies.

In conventional machine learning algorithms, most simulations were executed in the Matlab software environment, but the DL models are usually developed using Python programming language with numerous open-source toolboxes. The python language with more freely available DL toolboxes has helped the researchers to develop novel automated systems, and there is greater accessibility of computation resource to everyone thanks to cloud computing. Figure 1 shows that the TensorFlow and one of its high-level APIs, Keras, are widely used for epileptic seizure detection using DL in reviewed works due to their versatility and applicability.

**Figure 2:**
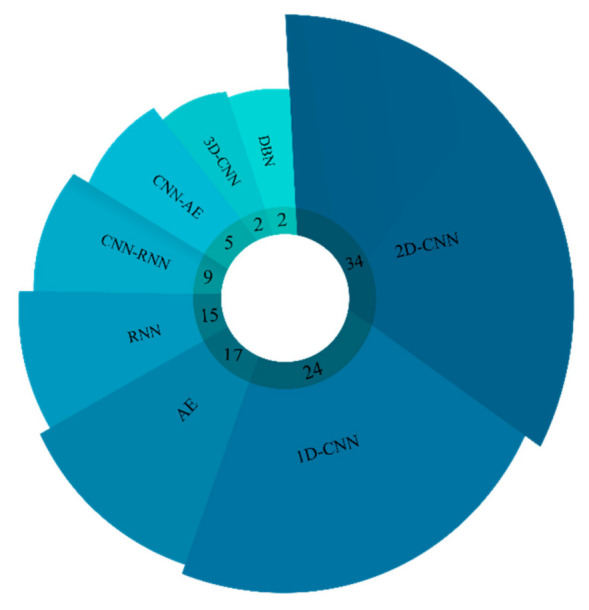
Number of studies conducted using various DL models from 2014 until now (2021).

Since 2016, substantial research has been done to detect epilepsy using DL models such as convolutional neural networks (CNNs), recurrent neural networks (RNNs), deep belief networks (DBNs), Autoencoders (AEs), CNN-RNNs, and CNN-AEs [30,31,32,33]. The number of studies in this area using DL is growing as new efficient models are proposed. Figure 2 provides the overview of number of studies conducted using various DL models from 2014 to 2021 in detecting epileptic seizures.

It can be noted from Figure 2 that various DL models have been exploited in the diagnosis of epileptic seizures. Compared to other DL techniques, 2D-CNN and 1D-CNN models are the most widely used in epileptic seizures detection. Researchers have mostly employed 2D-CNN models to diagnose epilepsy. In the diagnosis of epileptic seizures using 2D-CNN models, EEG signals are first converted into two-dimensional (2D) images using preprocessing methods such as short-time Fourier transform (STFT). Next, these images are applied to 2D-CNN networks. The second category comprises 1D-CNN models, which have achieved a special place among researchers for epileptic seizures detection. In this work, EEG signals are first preprocessed (noise removal and normalization) and then applied to 1D-CNN networks. Simple implementation and high efficiency are among the most important advantages of this type of network.

**Figure 3:**
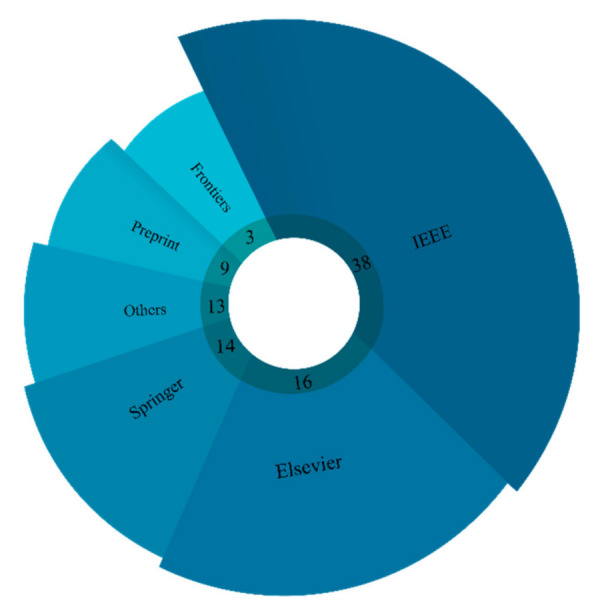
Search strategy used.

The keywords “EEG”, “MRI”, “Epilepsy”, “Epileptic Seizures”, and “Deep Learning” were used to search articles. These keywords were searched in various citation databases such as IEEE, Elsevier, Springer, Wiley, and ArXiv. Google Scholar was also used to search further. Figure 3 shows the number of accepted papers in each citation database. It is observed that the IEEE citation database contains the most accepted articles.

The main aims of this study are as follows:

- Providing information on available EEG datasets;
- Reviewing works done using various DL models for automated detection of epileptic seizures with various modality signals;
- Introducing future challenges on the detection of epileptic seizures;
- Analyzing the best performing model for various modalities of data.

Epileptic seizures detection using DL is discussed in Section 2. Section 3 describes the non-EEG-based epileptic seizure detection. Hardware used for epileptic seizures detection is provided in Section 4. Discussion on the paper is outlined in Section 5. The challenges faced by employing DL methods for epileptic seizure detection are summarized in Section 6. Finally, the conclusion and future work are delineated in Section 7.

## 2. Epileptic Seizures Detection Based on DL Techniques

**Figure 4:**
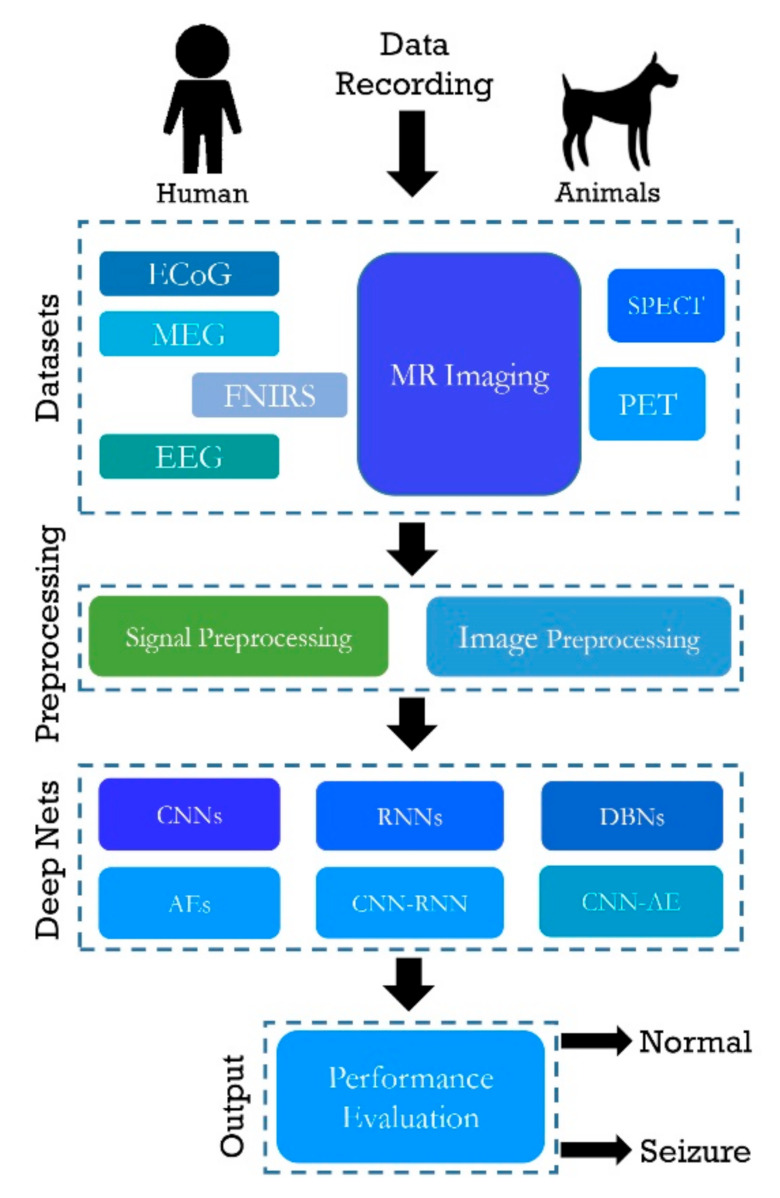
Block diagram of a DL-based CAD system for epileptic seizures.

Figure 4 illustrates the working of a computer-aided diagnosis system (CADS) for epileptic seizures using DL architectures. The input to the DL model can be EEG, MEG, ECoG, fNIRS, PET, SPECT, and MRI. Then, the signal is subjected to the preprocessing to remove the noise. These eliminated signals are used to develop the DL models. The performance of the model is evaluated using accuracy, sensitivity, and specificity. Additionally, a table combining all the works conducted on epileptic seizure detection using DL is presented in the table form in Appendix A of the paper.

### 2.1. Dataset

Datasets play an important role in developing accurate and robust CADS. Multiple EEG datasets, namely, Freiburg [34], CHB-MIT [35], Kaggle [36], Bonn [37], Flint-Hills [26], Bern-Barcelona [38], Hauz Khas [26], and Zenodo [39], are available to develop the automated epileptic seizure detection systems. The signals from these datasets are recorded either intracranial or from the scalp of humans or animals.

#### 2.1.1. Fribourg

The EEG dataset contains invasive EEG signals from 21 patients suffering from re-fractory focal epilepsy which were recorded during pre-surgical epilepsy monitoring at the epilepsy center of the University Hospital Fribourg. To provide direct recording from focal area, reduction of artifacts and achieving higher Signal to Noise Ratio (SNR), the in-tra-cortical grid, strip, and depth electrodes were used. The EEG signals were recorded using 128-channel Neurofile NT system with 6 contacts electrodes (three focal and three extra focal) and digitized by a 16bits A/D with sample rate of 256 Hz. For each patient, there are ictal and interictal data, the former contains seizures with at least 50 minutes’ of the pre-ictal region and the latter contains about 24 h of EEG data without seizure [34].

#### 2.1.2. CHB-MIT

The database comprises 844 h of continuous recording of scalp EEG signals with 163 seizures from 23 children, recorded according to intentional 10–20 standard electrode positions and sampled at 256 samples per second. The inter-ictal region is defined as the period between at least 4 h before the onset seizure and 4 h after the seizure ended. There are two types of seizures, called combined and main seizures, available in this database. The former are multiple seizures close to each other, while the later are great seizures considered for prediction. Generally, the prediction is meaningful for patients having less than 10 seizures per day. In this database, there are sufficient data available (at least three main seizures and 3 h inter-ictal recording) from 13 patients [35].

#### 2.1.3. Kaggle

The database is the epileptic seizures prediction challenge of the American Epilepsy Society and contains intracranial EEG signals from five dogs and two patients with 48 seizures and 627 h total duration. The EEG signals of dogs were acquired by 16 implantable electrodes, which were sampled at 400 KHz, while the EEG signals from patient 1 and patient 2 were recorded using 15 deep and 24 subdural electrodes, respectively, with sample rate of 5 KHz. In this database, 10 min segments of pre-ictal and inter-ictal data are available, and for each seizure, six pre-ictal segments (with 10 s distance) up to five minutes before seizure onset are accessible. The inter-ictal segments are selected randomly at least one week before each seizure [36].

#### 2.1.4. Bonn

Bonn database consists of five datasets, A, B, C, D, and E, each containing 100 single-channel EEG signals of 23.6 s duration. The EEG signals were digitized at a sample rate of 173.61 Hz by 12-bit A/D converter. Datasets A and B have the normal signals of five volunteers with eyes opened and closed states, respectively. The EEG signals of datasets C and D are related to pre-ictal region and were recorded from epileptogenic and left area of hippocampus, respectively. The EEG signals of E dataset are related to ictal region. Signals of datasets A and B were recorded using 10–20 scalp EEG standard, while the signals of C and D were intracranial EEG recorded using depth electrodes, and the signals of E were provided using both depth and strip electrodes. Depth electrodes are located symmetrically on hippocampus, while strip electrodes are located on lateral and base sections of neo cortex [37].

#### 2.1.5. Flint-Hills

The database presents electrocardiography signals with total duration of 1419 h and sample rate of 249 Hz. In addition, meta information about 59 seizures and information related to the position of electrodes are presented. The signals of this database were obtained using 48 to 64 electrodes for each patient [26].

#### 2.1.6. Bern Barcelona

Barcelona database was collected from the brain department of Bern Hospital of Barcelona and contains intracranial EEG of patients with focal epilepsy. Subjects were monitored for several days, and no antiepileptic drugs were used to determine seizures and possible surgery. The signals were acquired using AD-Tech intracortical electrodes, and one extra reference electrode based on 10–20 standard between PZ and FZ positions was used. The database contained two types of EEG signals: focal and extra focal EEG signals. Every dataset contained 3750 pairs of simultaneous recorded signals with duration of 20 s and sample rate of 512 Hz. The database consists of total 83 h EEG data from five patients with different ages [38].

#### 2.1.7. Hauz Khas

The database was collected at a brain center in Delhi, India and comprises of scalp EEG signals of 10 patients, recorded with AS40 system and sampled at a rate of 200 Hz in Hauz Khas neurons. The signals were filtered using band-pass filter with pass frequency of 0.5–70 Hz and classified as pre-ictal, inter-ictal, and ictal classes by neurologist experts [26].

#### 2.1.8. Zenodo

This dataset contains multichannel EEG recordings of 79 human neonates collected in Helsinki University Hospital, with the median recording duration of 74 min. The EEG data were annotated by three experts, and every expert has annotated about 460 seizures, 39 neonates had seizure and 22 neonates were seizure-free in consensus [26].

**Figure 5:**
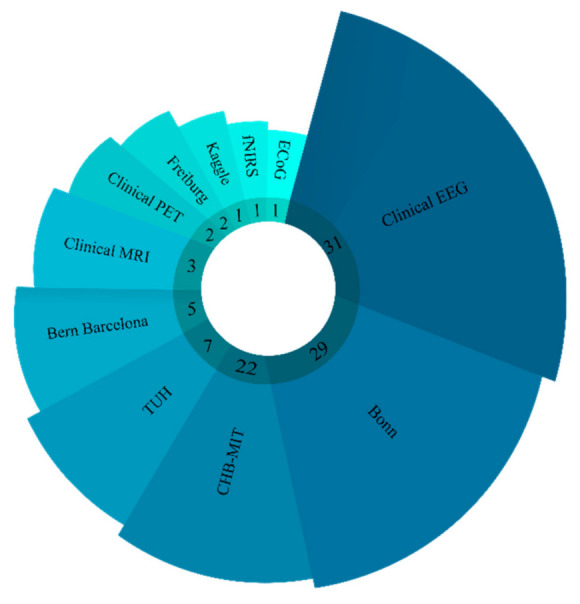
Usage of various datasets for automated detection of seizure using DL techniques by various studies.

The supplementary information on each dataset is listed in Table 1. Figure 5 shows the number of times each dataset employed epileptic seizures detection using DL techniques. It can be observed that the Bonn dataset is most widely used for automated detection of seizure using DL methods.

### 2.2. Preprocessing

In developing CADS using DL models with EEG signals, the preprocessing involves three steps: noise removal, normalization, and signal preparation for DL network applications [29,40]. In the noise removal step, finite impulse response (FIR) or infinite impulse response (IIR) filters are usually used to eliminate extra signal noise. Normalization is then performed using various schemes such as the z-score technique. Finally, different time domain, frequency, and time–frequency methods are employed to prepare the signals for the deployment of deep networks.

### 2.3. Review of Deep Learning Techniques

In contrast to conventional neural networks, or so-called shallow networks, deep neural networks are structures with more than two hidden layers. This increase in the size of the networks results in a massive rise in the number of parameters of the network, requiring appropriate methods for learning, and also measures to avoid overfitting of the learned network. Convolutional networks use filters convolved with input patterns instead of multiplying a weight vector (matrix), which reduces the number of trainable parameters dramatically.

Furthermore, other methods are suggested to help the network to learn, as well [41]. Pooling layers reduce the size of the input pattern to the next convolutional layer. Batch normalization, dropout, early stopping, unsupervised or semi unsupervised learning, and regularization techniques prevent the learned network from overfitting and increase the learning ability and speed. The AE and DBN are employed as unsupervised learning and then fine-tuned to avoid overfitting for limited labeled data. Long short-term memory (LSTM) and gated recurrent units (GRU) are RNNs capable of revealing the long-term time dependencies of data samples.

#### 2.3.1. Convolutional Neural Networks (CNNs)

CNNs are one class of the most popular DL networks to which most of the researches in machine learning have been devoted [30]. They were initially presented for image-processing applications, but have recently been adopted to one- and two-dimensional architectures for diagnosis and prediction of diseases using biological signals [42]. This class of DL networks is widely used for the detection of epileptic seizures using EEG signals. In two-dimensional convolutional neural networks (2D-CNN), the one-dimensional (1D) EEG signals are first transformed into two-dimensional plots by employing visualization methods such as spectrogram [43], higher-order bispectrum [44,45], and wavelet transforms, and are then applied to the input of the convolutional network. In 1D architectures, the EEG signals are applied in the one-dimensional form to the input of convolutional networks. In these networks, changes are made to the core architecture of 2D-CNN that makes it capable of processing the 1D-EEG signals. Therefore, since both 2D and one-dimensional convolutional neural networks (1D-CNNs) are used in the field of epileptic seizures detection, they are investigated separately.

##### A. 2D Convolutional Neural Networks (2D-CNNs)

**Figure 6:**
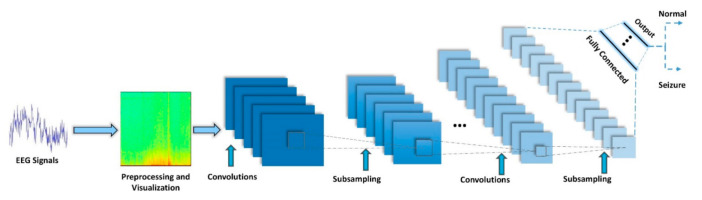
A typical 2D-CNN for epileptic seizure detection.

Nowadays, deep 2D networks are used for various medical applications such as diagnosis of COVID-19 in CT and X-ray [46,47], and autism spectrum disorders from MRI modalities [48]. First, in 2012, Krizovsky et al. [49] suggested this network to solve image classification problems, and then quickly used similar networks for different tasks such as medical image classification, in an effort to obviate the difficulties of previous networks and solve more intricate problems with better performance. Figure 6 shows a general form of a 2D-CNN used for epileptic seizure detection. The application of 2D-CNN architectures is arguably the most important architecture in the deep neural nets. More information about visualization and preprocessing method can be found in Appendix A.

In one study [50], the SeizNet 16-layer convolutional network is introduced, with additional dropout layers and batch normalization (BN) behind each convolutional layer having a structure similar to that of VGG-Net. The authors in [51] presented a new 2D-CNN model that can extract the spectral and temporal characteristics of EEG signals and used them to learn the general structure of seizures. Zuo et al. [52] developed the diagnosis of higher-frequency oscillations (HFO) epilepsy from 16-layer 2D-CNN and EEG signals. A DL framework called SeizureNet that uses convolution layers with dense connections is proposed in [53]. A novel DL model called the temporal graph convolutional network (TGCN) has been introduced by Covert et al. [54], comprising of five architectures with 14, 18, 22, 23, and 26 layers. Bouaziz et al. [55] split the EEG signals of CHB-MIT with 23 channels into 2 s time windows and then converted them into density images (spatial representation), which were fed as inputs to the CNN network.

##### B. AlexNet

FeiFei Li, Professor of Stanford University, created a dataset of labeled images of real-world objects and termed her project as ImageNet [56]. ImageNet organizes a computer vision competition called ILSVRC annually to solve the image classification problems. Alex Krizhevsky revolutionized the image classification world with his algorithm, AlexNet, which won the 2012 ImageNet challenge and started the whole DL era [49]. AlexNet won the competition by achieving the top-5 test accuracy of 84.6%. Taqi et al. [57] used the AlexNet network to diagnose focal epileptic seizures. This proposed network used the feature extraction approach and eventually applied the Softmax layer for classification purposes and achieved 100% accuracy. In another study, the AlexNet network was employed [58]. They transformed the 1D signal to 2D image by passing through the Signal2Image (S2I) module. The several methods used in this are signal as image, spectrogram, one-layer 1D-CNN, and two-layer 1D-CNN.

##### C. VGG

A research team at Oxford proposed the visual geometry group (VGG) model in 2014 [59]. They configured various models, and one such model was VGG-16, which was submitted to the ILSVRC 2014 competition. The VCG-16 comprises 16 layers and delivered an excellent performance for image classification problems. Ahmedt-Aristizabal et al. [60] performed VGG-16 architecture to diagnose epilepsy from facial images. Their proposed approach attempted to extract and classify semiological patterns of facial states automatically. After recording the images, the proposed VGG architecture is trained primarily by well-known datasets, followed by various networks such as 1D-CNN and LSTM in the last few layers. In [58], the VGG network used one-dimensional and two-dimensional signals. To train the models, Adam’s optimizer and a cross-entropy error function were used. They used the batch size and number of epochs as 20 and 100, respectively. The idea of detecting epileptic seizures on the sEEG signal plots was examined by Emami et al. [61]. In the preprocessing step, the signals were segmented into different time windows and VGG-16 was used for classification, using small (3 × 3) convolution filters to efficiently detect small EEG signal changes. This architecture was pre-trained by applying an ImageNet dataset to differentiate 1000 classes, and the last two layers had 4096 and 1000 dimensional vectors. They modified these last two layers to have 32 and 2 dimensions, respectively, to detect seizure and non-seizure classes.

##### D. GoogleNet

GoogLeNet won the 2014 ImageNet competition with 93.3% top-5 test accuracy [62]. This 22-layer network was called GoogLeNet to honor Yann Lecun, who designed LeNet. Before the introduction of GoogLeNet, it was stated that by going deep, one could achieve better accuracy and results. Nevertheless, the google team proposed an architecture called inception, which achieved better performance by not going deep but by better design. It represented a robust design by using filters of different sizes on the same image. In the field of EEG signal processing to diagnose epileptic seizures, this architecture has recently received the attention of researchers. Taqi et al. [57] used this network in their preliminary studies to diagnose epileptic seizures. Their model was used to extract features from the Bern-Barcelona dataset and achieved excellent results.

##### E. ResNet

**Figure 7:**
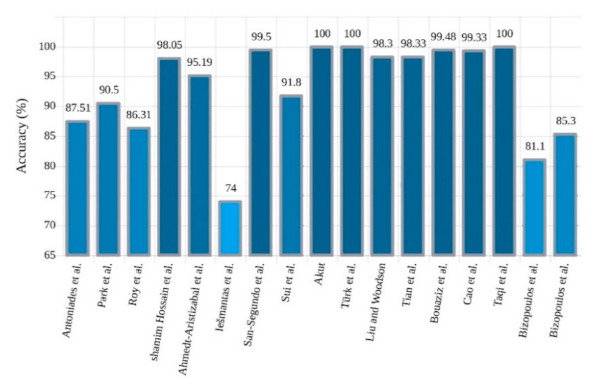
Sketch of accuracy (%) obtained by various authors using 2D-CNN models for seizure detection.

Microsoft’s ResNet won the ImageNet challenge with 96.4% accuracy by applying a 152-layer network that utilized a ResNet module [63]. In this network, residual blocks capable of training deep architecture were introduced by using skip connections that copied inputs of each layer to the next layer. The idea was to learn something different and new in the next layer. So far, little research has been accomplished on the implementation of ResNet networks to diagnose epilepsy, but this may grow significantly in the coming days. Bizopoulos et al. [58] introduced two ResNet and DenseNet architectures to diagnose epileptic seizures and attained good results. They showed that S2I-DenseNet based model with an average of 70 epochs was sufficient to gain the best accuracy of 85.3%. A summary of related works done using 2D-CNNs is shown in Table 2. A sketch of accuracy accuracy (%) obtained by various authors is shown in Figure 7.

##### F. 1D—Convolutional Neural Network (1D-CNN)

**Figure 8:**
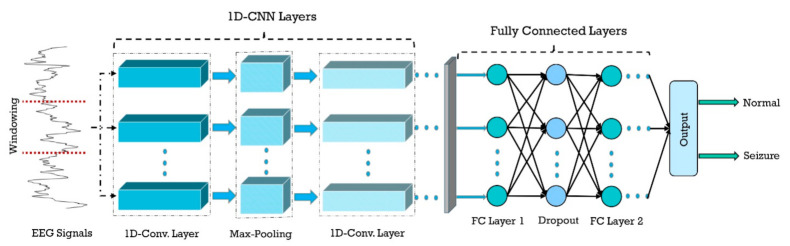
Typical sketch of the 1D-CNN model that can be used for epileptic seizure detection.

1D-CNNs are intrinsically suitable for processing of biological signals such as EEG for epileptic seizures detection [2]. These architectures present a more straightforward structure, and a single pass of them is faster as compared with CNN with 2D architecture, due to fewer parameters. The most important superiority of 1D to 2D architectures is the possibility of employing pooling and convolutional layers with a larger size. In addition to that, signals are 1D in nature, and using preprocessing methods to transform them to 2D may lead to information loss. Figure 8 shows a general form of a 1D-CNN used for epileptic seizure detection.

**Figure 9:**
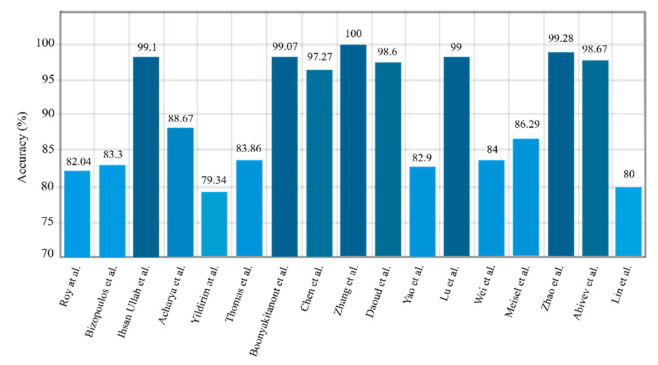
Sketch of accuracy (%) versus authors obtained using 1D-CNN models for seizure detection.

The authors in [58] conducted experiments using 1D-LeNet, AlexNet, VGGnet, ResNet, and DenseNet architectures, and applied well-known 2D architectures in 1D space in the first study in this section. In [80], 1D-CNN was used for feature extraction procedure. The researchers in [81] used 1D-CNN for other work. They used a CHB-MIT dataset, and the signals from each channel were segmented into 4 s intervals; overlapping segments were also accepted to increase the data and accuracy. Combining CNNs with conventional feature extraction methods was explored in [82]; they used the empirical mode decomposition (EMD) method for feature extraction, and CNN was used to acquire high accuracy in the multiclass classification tasks. In [83], a framework for the diagnosis of epileptic seizures is presented that combined the capability of interpreting probabilistic graphical models (PGMs) with advances in DL. The authors in [84] submitted a 1D-CNN architecture-defined CNN-BP (standing for CNN bipolar). In this work, they used the data from patients monitored with combined foramen ovale (FO) electrodes and EEG surface electrodes. A new scheme to classify EEG signals based on temporal convolution neural networks (TCNN) was introduced by Zhang et al. [85]. Table 3 shows the summary of related works done using 1D-CNNs. Figure 9 shows the sketch of accuracy (%) obtained by various authors using 1D-CNN models for epileptic seizures detection.

#### 2.3.2. Recurrent Neural Networks (RNNs)

**Figure 10:**
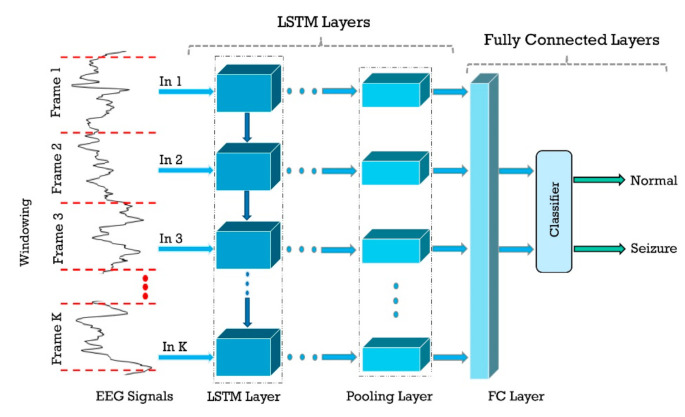
Sample RNN model that can be used for seizure detection.

Sequential data such as text, signals, and videos show characteristics such as variable and great length, which makes them not suitable for simple DL methods [41]. However, these data form a significant part of the information in the world, compelling the need for DL-based schemes to process these types of data. RNNs are the solution suggested to overcome the mentioned challenges, and are widely used for physiological signals. Figure 10 shows a general form of RNN used for epileptic seizure detection. In the following section, an overview of popular RNN models are presented in addition to the reviewed papers.

##### A. Long Short-Term Memory (LSTM)

The main problem of a simple RNN is short-term memory. RNN may leave out key information as it has a hard time transporting information from earlier time steps to the next steps in long-sequence data. Another drawback of RNN is the vanishing gradient problem [30,31,32,33]. The problem arises because of the shrinking of gradients as it back-propagates. To solve the short-term memory problem, LSTM gates were created [30]. The flow of information can be regulated through gates. The gates can preserve the long sequence of necessary data, and throw away the undesired ones. The building block of LSTM is the cell state and its gates.

In this section, Golmohammadi et al. [68] evaluated two LSTM architectures with three and four layers together with the Softmax classifier in their investigation and obtained satisfactory results. In [92], three-layer LSTMs are used for feature extraction and classification. The sigmoid active function is used in the last fully connected (FC) layer for classification. According to directed experiments in [98], they employed two architectures: LSTM and GRU. The LSTM GRU model architecture is composed of a layer of Reshape, four layers of LSTM/GRU with the activator, and one layer of FC with sigmoid activator. In another work, Yao et al. [102] practiced ten different and independently ameliorated RNN (IndRNN) architectures and achieved the best accuracy using Dense IndRNN with attention (DIndRNN) with 31 layers.

##### B. Gated Recurrent Unit (GRU)

**Figure 11:**
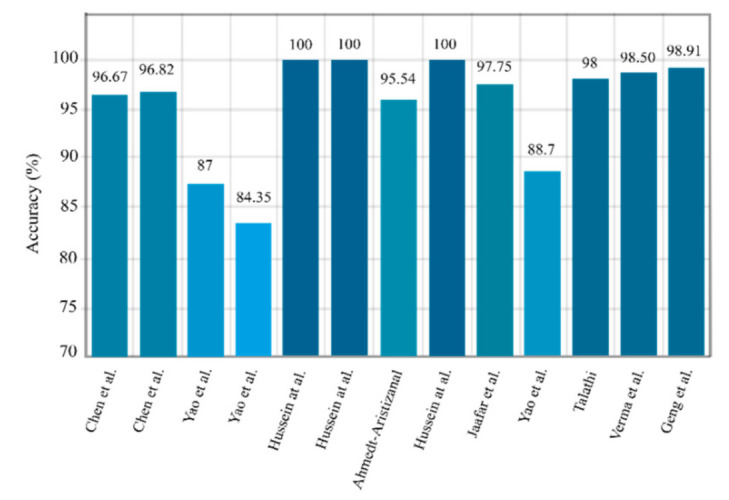
Sketch of accuracy (%) obtained by authors using RNN models for seizure detection.

One variation of LSTM is GRU, which combines the input and forgets gates into one update gate [30,31,32,33]. It merges the input and forgets gates and also makes some other modifications. The gating signals are decreased to two. One is the reset gate, and another is the updating gate. These two gates decide which information is necessary to pass to the output. In one experiment, Chen et al. [92] used a three-layer GRU network with sigmoid classifier and yielded 96.67% accuracy. Talathi et al. have used a new CADS based on GRU for epileptic seizure detection [103]. In the proposed method, during the preprocessing, the input signals are split into time windows and spectrogram are obtained from them. Then, these plots are fed to a four-layer GRU network with a Softmax FC layer in the classification stage; 98% accuracy was achieved. In another study, Roy et al. [104] employed a five-layer GRU network with Softmax classifier and achieved remarkable results. Table 4 provides the summary of related works done using RNNs. Figure 11 shows the sketch of accuracy (%) obtained by various authors using RNN models for seizure detection.

#### 2.3.3. Autoencoders (AEs)

**Figure 12:**
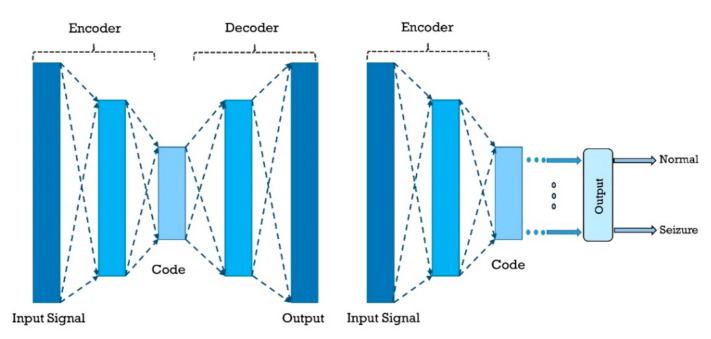
Sample AE network that may be used for seizure detection.

AE is an unsupervised machine learning model for which the input is the same as output [30,31,32,33]. Input is compressed to a latent-space representation, and then the output is obtained from the representation. Therefore, in AE, the compression and decompression functions are coupled with the neural network. AE consists of three parts, i.e., encoder, code, and decoder. AE networks are most commonly used for feature extraction or dimensionality reduction in the brain signal processing. Figure 12 shows a general form of an AE used for epileptic seizures detection. As the first research in this section, Rajaguru et al. [113] separately surveyed the multilayer AE (MAE) and expectation-maximization with principal component analysis (EM-PCA) methods to diminish the representation dimensions and then employed the GA for classification. They obtained an average classification accuracy of 93.78% when MAEs were applied for dimensionality reduction and combined with GA as classifier. In another work, it was proposed to design an automated system based on AEs for the diagnosis of epilepsy using the EEG signals [114]. First, Harmonic wavelet packet transform (HWPT) was used to decompose the signal into frequency sub-bands, and then fractal features, including box-counting (BC), multiresolution BC (MRBC), and Katz fractal dimension (KFD), were extracted from each of the sub-bands.

##### A. Other Types of AEs

To create a more robust representation, a number of schemes such as denoising AE (DAE) (which tries to recreate input from a corrupted form of it) [41], stacked AE (SAE) (stacking a few AEs on top of each other to go deeper) [41], and sparse AEs (SpAE) (which attempts to harness from sparse representations) [41] have been applied. These methods might pursue other objectives as well, for example, the DAE can be used to recover the corrupted input.

Works in this section begin with Golmohammadi et al. [68], who presented various deep networks, one of which is stacked denoising AE (SDAE). Their architecture in this section consists of three layers, and the final results demonstrated good performance of their approach. Qiu et al. [115] exerted the windowed signal, z-score normalization step of preprocessing EEG signals and imported preprocessed data into the denoising sparse AE (DSpAE) network. In their experiment, they achieved an outstanding performance of 100% accuracy. In [116], a high-performance automated EEG analysis system based on principles of machine learning and big data is presented, which consists of several parts. At first, the signal features are extracted by linear predictive cepstral coefficients (LPCC) coefficients, then three paths are applied for precise detection. The first pass is sequential decoding using hidden Markov models (HMMs), the second pass is composed of both temporal and spatial context analysis based on DL, and in the third pass, a probabilistic grammar is employed.

**Figure 13:**
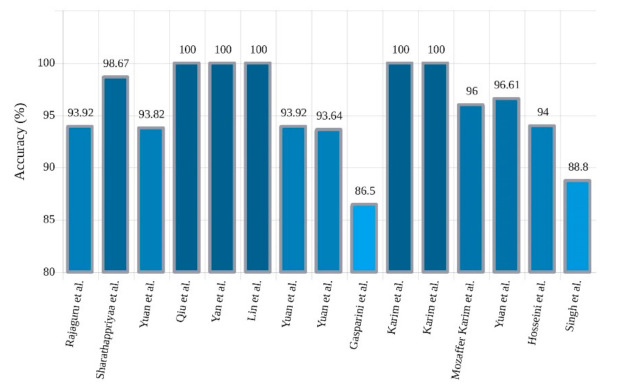
Sketch of accuracy (%) versus authors obtained using AE models for seizure detection.

In another study, Yan et al. [117] proposed a feature extraction and classification method based on SpAE and support vector machine (SVM). In this approach, first, the feature extraction of the input EEG signals is performed using SAE, and, finally, the classification is performed by SVM. Another SAE architecture was proposed by Yuan et al. [118], which is namedWave2Vec. In the preprocessing stage, the signals were first framed, and in the deep network segment, the SAE with Softmax was applied and achieved 93.92% accuracy. Following the experiments of Yuan et al., in [119], different stacked sparse denoising AE (SSpDAE) architectures have been tested and compared. In this work, feature extraction is accomplished by the SSpDAE network and finally classification by Softmax. They obtained an accuracy of 93.64%. Table 5 provides the summary of related works done using AEs. In addition, Figure 13 shows the comparison of the accuracies obtained by different researchers.

#### 2.3.4. Deep Belief Networks (DBNs)

Restricted Boltzmann machines (RBM) is a variant of deep Boltzmann machines (DBM) and an undirected graphical model [30]. The unrestricted Boltzmann machines may also have connections between the hidden units. Stacking the RBMs forms a DBN; RBM is the building block of DBN. DBNs are unsupervised probabilistic hybrid generative DL models comprising latent and stochastic variables in multiple layers [30,31,32,33]. Furthermore, a variation of DBN is called convolutional DBN (CDBN), which could successfully scale the high-dimensional model and uses the spatial information of the nearby pixels [30,31,32,33]. DBNs are probabilistic, generative, unsupervised DL models which contain visible and multiple layers of hidden units [30,31,32,33]. Xuyen et al. [129] used DBN to identify epileptic spikes in EEG data. The proposed architecture in their study consisted of three hidden layers and achieved an accuracy of 96.87%. In another study, Turner et al. [130] applied the DBN network to diagnose epilepsy and found promising results.

#### 2.3.5. Convolutional Recurrent Neural Networks (CNN-RNNs)

The highly efficient combination of DL networks used to predict and detect epileptic seizures from EEG signals is the CNN-RNN architecture. Adding convolutional layers to RNN helps to find spatially nearby patterns effectively as RNN characteristic is more suitable for time-series data. In [68], they applied numerous preprocessing schemes; then, a modified CNN-LSTM architecture was proposed comprising 13 layers and the sigmoid was used for the last layer. Finally, the proposed approach demonstrated better performance.

Roy et al. [69] used different CNN-RNN hybrid architectures to improve the experimental results. Their first network comprised a one-dimensional seven-layer CNN-GRU convolution architecture, and the second one is a three-dimensional (3D) CNN-GRU network. In another work, Roy et al. [104] concentrated on natural and abnormal brain activities and suggested four different DL architectures. The proposed ChronoNet model was developed using previous models. It achieved 90.60% and 86.57% training and test accuracies, respectively.

**Figure 14:**
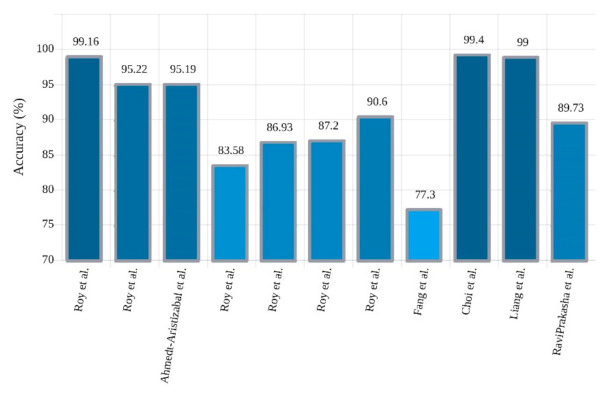
Sketch of accuracy (%) versus different researchers obtained using CNN-RNN models for seizure detection.

Fang et al. [131] used the Inception-V3 network. At the outset, a preliminary training was used on this network. Then, to fine-tune this architecture, an RNN- based network called spatial temporal GRU (ST-GRU) was applied, and achieved 77.30% accuracy. Choi et al. [132] proposed a multiscale 3D-CNN with RNN model for the detection of epileptic seizures. The CNN module output is applied as the input of the RNN module. The RNN module consists of a unilateral GRU layer that extracts the temporal feature of epileptic seizures, which are finally classified using an FC layer. At the end of this section, generalized information from the CNN-RNN research is presented in Table 6 and Figure 14, respectively.

#### 2.3.6. Convolutional Autoencoders (CNN-AEs)

**Figure 15:**
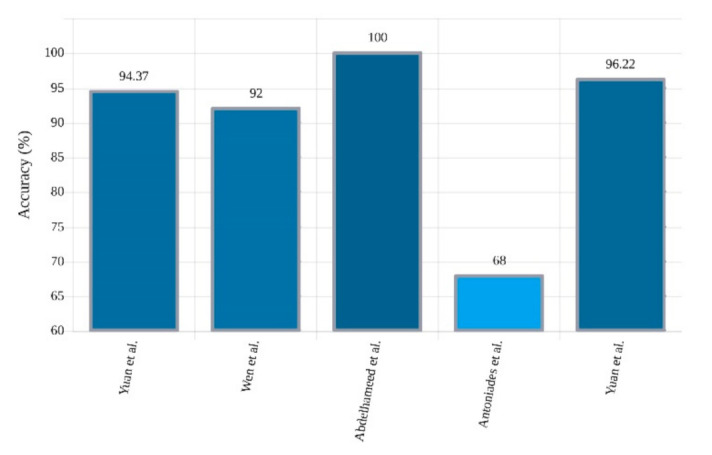
Sketch of accuracy (%) versus authors obtained using CNN-AE models for seizure detection.

In addition to finding nearby patterns, convolutional layers can reduce the number of parameters in structures such as AEs. These two reasons make their combination suitable for many tasks such as unsupervised feature extraction for epileptic seizure detection. A novel approach based on CNN-AE was presented by Yuan et al. [136]. At the feature extraction stage, two deep approaches, AE and 2D-CNN, were used to extract the supervised and unsupervised features, respectively. The unsupervised features were obtained directly from the input signals, and the supervised features were acquired from the spectrogram of the signals. Finally, the Softmax classifier was utilized for classification and achieved 94.37% accuracy. In another investigation, Yuan et al. [137] proposed an approach called deep fusional attention network (DFAN), which can extract channel-aware representations from multichannel EEG signals. They developed a fusional attention layer that utilized a fusional gate to fully integrate multiview information to quantify the contribution of each biomedical channel dynamically. A multiview convolution encoding layer, in combination with CNN, has also been used to train the integrated DL model. Table 7 provides the summary of related works done using CNN-AEs, and Figure 15 shows the accuracies (%) obtained by different researchers.

## 3. Non-EEG-Based Epileptic Seizures Detection

### 3.1. Medical Imaging

Various DL models were developed to detect epileptic seizure using sMRI, fMRI, and PET scans with or without EEG signals [141,142,143,144,145,146,147,148]. These models outperformed the conventional models in terms of automatic detection and monitoring of the disease. However, due to the nature and difficulties in using imaging methods, these models are mostly practiced for localization and detection of seizure.

The authors of [141] proposed automatic localization and detection of focal cortical dysplasia (FCD) from the MRI modality using a CNN model. The diagnosis of FCD rate is only 50% despite the progress in the analytics of MRI modalities. Gill et al. [142] proposed a CNN-based algorithm with feature learning capability to detect FCD automatically. The authors [143] designed DeepIED based on DL and EEG-fMRI scans for epilepsy patients, combining the general linear model with EEG-fMRI techniques to estimate the epileptogenic zone. Hosseini et al. [144] proposed an edge computing autonomic framework for evaluation, regulation, and monitoring of epileptic brain. The epileptogenic network estimated the epilepsy using rs-fMRI and EEG. Shiri et al. [148] presented a technique for direct attenuation correction of PET images by applying emission data via CNN-AE. Nineteen radiomic features from 83 brain regions were evaluated for image quantification via Hammersmith atlas. Finally, the summary of related works done using medical imaging methods and DL is shown in Table 8.

### 3.2. Other Neuroimaging Modalities

Ravi Prakash et al. [135] introduced an algorithm based on DL for ECoG-based functional mapping (ECoG-FM) for eloquent language cortex identification. However, the success rate of ECoG-FM is low as compared with electro-cortical stimulation mapping (ESM). In another work, Rosas-Romero et al. [149] have used fNIRS to detect epileptic seizure and obtained better performance than achieved using conventional EEG signals.

## 4. Rehabilitation Systems for Epileptic Seizures Detection

**Figure 16:**
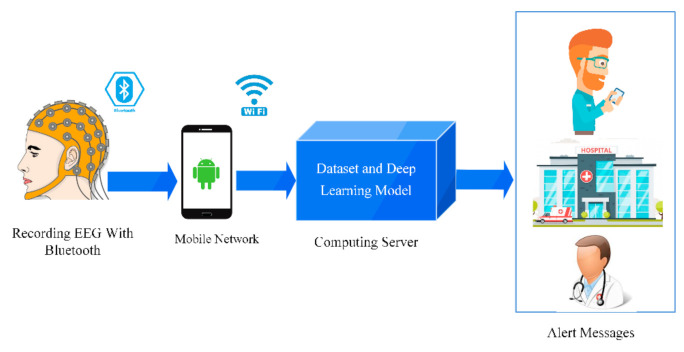
Block diagram of proposed epileptic seizure detection system using DL methods with EEG signals.

The high performance and robustness to noise have made the DL techniques suitable for commercial products. Nowadays various commercial products have been developed in the field of DL, one of which is DL applications and hardware for diagnosing epileptic seizures. In the first study investigated, the brain–computer interface (BCI) system was developed using an AE for epileptic seizure detection by Hosseini et al. [127]. In another study, Singh et al. [128] indicated a utilitarian product for the diagnosis of epileptic seizures, which comprised the user segment and the cloud segment. The block diagram of the proposed system presented by Singh et al. is shown in Figure 16.

Kiral-Kornek et al. [150] demonstrated that DL in combination with neuromorphic hardware could help in developing a wearable, real-time, always-on, patient-specific seizure warning system with low power consumption and reliable long-term performance.

## 5. Discussion

Nowadays, many people worldwide have epileptic seizures and suffer from these neurological disorders. Early detection of epileptic seizures is of substantial importance because it directly affects the patients’ quality of life and can enhance their self-confidence at all stages of life. So far, much research has been accomplished to diagnose epileptic seizures using AI techniques. The objective of these studies is to assist physicians in accurate epileptic seizures diagnosis. AI research involves conventional machine learning [151] and DL [152,153,154,155,156] scopes. Until recently, many machine learning methods that were adopted to automatically detect seizures could not be seriously used for a variety of real-time diagnostic aid tools for epileptic seizures due to their disadvantages. DL is one of the state-of-the-art fields of epileptic seizure detection that has been employed for epileptic seizure detection since 2016. In recent years, the research growth in epileptic seizure diagnosis using DL is proceeding rapidly due to the simultaneous development of DL toolboxes as well as graphics processing units (GPUs). Applying DL techniques to diagnose epileptic seizures gives doctors hope that in the not-too-distant future a variety of rehabilitation tools will be developed for patients with epileptic seizures. Table A1 in the Appendix shows the overview of works done in this area. It also shows the type of dataset used, implementation tool, preprocessing, DL network, and evaluation methods utilized.

As shown in this study, various DL structures are applied for epileptic seizure detection, yet none of them has superiority over others. The best structure should be chosen carefully based on the dataset and problem characteristics, such as the need for real-time detection or minimum acceptable accuracy or even the use of pre-trained models. There are many databases available with different models. Hence, it is difficult to compare them as they have been developed using different datasets and models. Overall, one of the most important advantages of DL algorithms is their high performance. Hence, such models have been widely used for many applications. Another advantage of DL methods is that they are robust to noise. Therefore, noise removal can be omitted in many applications. However, they need more data to train, and training takes time. Developing a robust model is time consuming and requires huge data.

## 6. Challenges

There are several challenges in diagnosing epileptic seizures using neuroimaging modalities and DL procedures. Inaccessibility of datasets with high registration time is the first challenge in this area. The datasets available for diagnosing epileptic seizures have a finite registration time (or recording), making it difficult to conduct serious (or important) research in the field of epileptic seizures. The complete datasets are not shared in the public domain, only a portion of the data may be available. Hence, real-time diagnosis of epileptic seizures is still challenging. However, research in the field of real-time epileptic seizures diagnosis has been performed, using clinical data [157,158,159].

Due to the lack of accessible datasets, researchers have not yet been able to present a DL-based CADS for diagnosing epileptic seizures with optimum performance. Additionally, it is not possible to combine the available EEG datasets to enhance the efficiency of DL networks. This is because each of the datasets presented possesses different sampling frequencies, and in order to achieve higher detection accuracy, it is not pragmatic to integrate them to feed to DL networks.

Table 1 shows all available EEG datasets used for epileptic seizure detection. However, other neuroimaging modalities such as MRI are used for epileptic seizures detection. In [141,142,143,144,145,146,147,148], MRI modalities coupled with DL methods have been used to diagnose epileptic seizures. Datasets with non-MRI modalities are not available, and this has led to limited research in this area. Therefore, providing datasets from other neuroimaging modalities is important to conduct research.

Nowadays, DL models have made considerable advancements [160,161,162,163,164]. This has resulted in the development of computer hardware [165,166] that is expensive and not easily accessible to the researchers. Researchers working in the field of epileptic seizures detection/prediction do not always have access to high-power hardware to implement novel DL models. Although powerful computing servers are available by Google, constraints such as the amount of data that can be uploaded to these servers and execution time are still the challenges.

## 7. Conclusion and Future Works

In recent years, a lot of research has been done in the epileptic seizures detection field using artificial intelligence methods [167,168,169,170,171,172,173,174,175]. In this paper, a comprehensive review of works done in the field of epileptic seizure detection using various DL techniques such as CNNs, RNNs, and AEs is presented. Various screening methods have been developed using EEG and MRI modalities. We have investigated the epileptic seizures detection using DL-based practical and applied hardware methods. It is very encouraging that much of the future research will concentrate on hardware—practical applications aid in the accurate detection of such diseases. The functional hardware has also been utilized to boost the performance of detection strategies. Furthermore, the models can be placed in the cloud by hospitals. Therefore, handheld applications, mobile or wearable devices, may be equipped with such models, and cloud servers will perform the computations; by taking benefit from predictive models, these devices can be used to avert patients in a timely manner. Alert messages may be generated to the family, relatives, the concerned hospital, and doctor in the detection of epileptic seizures through the handheld devices or wearables, and thus the patient can be provided with proper treatment in time. Moreover, a cap with EEG electrodes in it can obtain the EEG signals, which can be sent to the model kept in the cloud to achieve real-time detection. Additionally, if we can detect early stage of seizure using interictal periods of EEG signals, the patient can take medication immediately and prevent seizure. This field of research requires more research that combines different screening methods for more precise and fast detection of epileptic seizures and also applies semi supervised and unsupervised methods to further overcome the dataset size limits. Finally, having publicly available comprehensive datasets can help to develop an accurate and robust model that can detect the seizure in the early stage.

## Figures and Tables

**Table 1:** Review of popular and available EEG datasets for epileptic seizures detection.

Dataset	Number of Patients	Number of Seizures	Recording	Times	Sampling Frequency
Flint-Hills [26]	10	59	Continues intracranial ling term ECoG	1419	249
Hauz Khas [26]	10	NA	Scalp EEG	NA	200
Freiburg [34]	21	87	IEEG	708	256
CHB-MIT [35]	22	163	Scalp EEG	844	256
Kaggle [36]	5 dogs	48	IEEG	627	400
	2 patients				5 KHz
Bonn [37]	10	NA	Surface and IEEG	39 m	173.61
Bern Barcelona [38]	5	3750	IEEG	83	512
Zenodo [39]	79 neonatal	460	Sclap EEG	74 m	256

**Table 2:** Summary of related works done using 2D-CNNs.

Works	Networks	Number of Layers	Classifier	Accuracy (%)
[50]	SeizNet	16	NA	NA
[51]	2D-CNN	9	Softmax	98.05
[52]	2D-CNN	16	Softmax	NA
[53]	SeizureNet	133	Softmax	NA
[54]	TGCN	14	Sigmoid	NA
		18		
		22		
		22		
		26		
[55]	2D-CNN	8	Softmax	99.48
[57]	GoogleNet	Standard Networks	Softmax	100
	AlexNet			
	LeNet			
[58]	Different PreTrain Networks	Standard Networks	Softmax	85.30
[60]	2D-CNN	VGG-16	SVM	95.19
		VGG-8		
[64]	2D-CNN	3	Logistic Regression (LR)	87.51
		4		
[65]	2D-CNN	9	Softmax	NA
[66]	Combination 1DCNN and 2D-CNN	11	Sigmoid	90.58
[67]	2D-CNN	18	Softmax	NA
[68]	2D-CNN/MLP hybrid	11	Sigmoid	NA
[69]	2D-CNN	9	Softmax	86.31
[70]	2D-CNN with 1D-CNN	12	Softmax	NA
[71]	2D-CNN	6	Softmax	74.00
[72]	2D-CNN	12	Softmax and Sigmoid	99.50
[73]	2D-CNN	16		91.80
[74]	2D-CNN	23	Softmax	100
[75]	2D-CNN	5	Softmax	100
[76]	2D-CNN	14	Softmax	98.30
[77]	2D-CNN	7	MV-TSK-FS	98.33
		5		
	3D-CNN	8		
[78]	2D-CNN	23	Sigmoid	NA
		18	RF	
[79]	2D-CNN	7	KELM	99.33
[61]	2D-CNN	VGG-16	Softmax	NA

**Table 3:** Summary of related works done using 1D-CNNs.

Works	Networks	Number of Layers	Classifier	Accuracy (%)
[58]	1D-CNN	VGG-16, 19	Standard PreTrain Nets	83.30
		DenseNet 161		
[69]	1D-CNN	7	Softmax	82.04
[80]	1D-CNN	5	Softmax, SVM	86.86
[81]	1D-CNN	33	NA	99.07
[82]	1D-CNN	12	Softmax	98.60
[83]	PGM-CNN	10	Softmax	NA
[84]	1D-CNN-BP	14	Sigmoid	NA
[85]	1D-TCNN	NA	NA	100
[86]	P-1D-CNN	14	Softmax	99.10
[87]	1D-CNN	13	Softmax	88.67
[88]	MPCNN	11	Softmax	NA
[89]	1D-FCNN	11	Softmax	NA
[90]	1D-CNN	5	Binary LR	NA
[91]	1D-CNN	23	Softmax	79.34
[92]	1D-CNN	4	Sigmoid	97.27
[93]	1D-CNN	13	NA	82.90
[94]	1D-CNN with residual connections	17	Softmax	99.00
				91.80
[95]	1D-CNN	15	Softmax	84.00
[96]	1D-CNN	10	Sigmoid	86.29
[97]	1D-CNN	13	Softmax	NA
[98]	1D-CNN	9	Sigmoid	NA
[99]	1D-CNN	8	NA	99.28
[100]	1D-CNN	15	Softmax	98.67
[101]	Deep ConvNet	14	Softmax	80.00

**Table 4:** Summary of related works done using RNNs.

Works	Networks	Number of Layers	Classifier	Accuracy (%)
[68]	LSTM	3	Sigmoid	NA
		4		
[92]	LSTM	3	Sigmoid	96.67
	GRU			96.82
[93]	IndRNN	48	NA	87.00
	LSTM	4		84.35
[98]	LSTM	6	Sigmoid	NA
	GRU			
[102]	ADIndRNN	31	NA	88.70
[103]	GRU	4	LR	98.00
[104]	GRU	5	Softmax	NA
[105]	RNN	NA	MLP	NA
[106]	LSTM	4	Softmax	100
[107]	LSTM	2	Sigmoid	95.54
		5		
[108]	LSTM	4	Softmax	100
[109]	LSTM	3	Softmax	97.75
[110]	LSTM	4	Softmax	100
[111]	GRU	3	LR	98.50
[112]	Bi LSTM	One Bi LSTM	Softmax	98.91

**Table 5:** Summary of related works done using AEs.

Works	Networks	Number of Layers	Classifier	Accuracy (%)
[68]	SDAE	3	NA	NA
[113]	MAE	NA	GA	93.92
[114]	AE	3	Softmax	98.67
[115]	DSpAE	3	LR	100
[116]	SPSW-SDA	Each Model has 3 hidden layers	LR	NA
	6W-SDA			
	EYEM-SDA			
[117]	SpAE	Single-Layer SpAE	SVM	100
[118]	Wave2Vec	NA	Softmax	93.92
	SSpDAE	2		93.64
[119]	SAE	3	Softmax	96.10
[120]	AE	One Layer	Sigmoid	NA
[121]	SSpDAE	8	Softmax	93.82
[122]	SSpAE	3	Softmax	100
[123]	SAE	3	Softmax	86.50
[124]	SSpAE	3	Softmax	100.00
[125]	SpAE	3	Softmax	100.00
[126]	SAE	3	Softmax	96.00
[127]	SSpAE	3	Softmax	94.00
[128]	SAE	3	Softmax	88.80

**Table 6:** Summary of related works done using CNN-RNNs.

Works	Networks	Number of Layers	Classifier	Accuracy (%)
[60]	2D CNN-LSTM	VGG-16	Sigmoid	95.19
[68]	2D-CNN BiLSTM	13	Sigmoid	NA
[69]	1D CNN-GRU	7	Softmax	99.16
	TCNN-RNN	10		95.22
[104]	C-RNN	8	Softmax	83.58
	IC-RNN	14		86.90
	C-DRNN	8		87.20
	ChronoNet	14		90.60
[131]	ST-GRU ConvNets	Inception-V3 + GRU	NA	77.30
[132]	3D-CNN BiGRU	NA	NA	99.40
[133]	2D CNN-LSTM	8	NA	NA
[134]	2D CNN-LSTM	18	Softmax	99.00
[135]	1D CNN-LSTM	7	Sigmoid	89.73
		8		

**Table 7:** Summary of related works done using CNN-AEs.

Works	Networks	Number of Layers	Classifier	Accuracy (%)
[136]	CNN-AE	10	Softmax	94.37
[137]	CNN-AE	NA	Softmax	96.22
[138]	CNN-AE	15	Different Classifiers	92.00
[139]	1D-CNN-AE	16	Sigmoid	100
[140]	CNN-ASAE	8	LR	66.00
	CNN-AAE	7		68.00

**Table 8:** Summary of related works done using MRI modalities and DL.

Works	Networks	Number of Layers	Classifier	Accuracy (%)
[141]	2D-CNN	30	sigmoid	82.50
[142]	2D-CNN	11	Softmax	NA
[143]	ResNet	31	Softmax	NA
			Triplet	
[144]	2D-CNN	NA	SVM	NA
[145]	2D-CNN	11	Softmax	89.80
	3D-CNN			82.50
[146]	2D-CNN	NA	NA	NA
[147]	ResNet	14	sigmoid	98.22
	VGGNet			
	Inception-V3			
	SVGG-C3D			
[148]	Deep Direct Attenuation Correction (Deep-DAC)	44	Tanh	NA

## Data Availability

Not applicable.

## Appendix A

Table A1 shows the detailed summary of DL methods employed for automated detection of epileptic seizures.

**Table A1:** Summary of DL methods employed for automated detection of epileptic seizures.

Work	Dataset	Preprocessing	DL Toolbox	DL Network	K-Fold	Classifier	Accuracy (%)
[50]	Clinical	Down-Sampling, Normalization, Data Augmentation	Keras	SeizNet	--	--	--
[51]	CHB-MIT	Visualization	PyTorch	2D-CNN	--	Softmax	98.05
[52]	Clinical	Filtering, Normalization, Visualization	NA	2D-CNN	10	Softmax	NA
[53]	TUH	DivSpec	PyTorch	SeizureNet	5	Softmax	NA
[54]	Clinical	STFT	NA	TGCN	--	Sigmoid	NA
[55]	CHB-MIT	Spatial Representation	NA	2D-CNN	--	Softmax	99.48
[61]	Clinical	Filtering, Visualization	Chainer	2D-CNN	--	Softmax	NA
[64]	Clinical	Spectrogram	NA	2D-CNN	--	LR	87.51
[65]	Clinical	Normalization	Matlab	2D-CNN	--	Softmax	NA
[66]	Clinical	Filtering	NA	1D-CNN with 2D-CNN	--	Sigmoid	90.50
	CHB-MIT						85.60
[67]	Clinical	Filtering, Down-Sampling	Octave	2D-CNN	--	Softmax	NA
			Keras				
			Theano				
[68]	TUH	Filtering	NA	CNN-RNN	--	Different Methods	NA
	Clinical						
[69]	TUH	Different Methods	NA	1D-CNN-GRU	--	Softmax	99.16
[70]	Clinical	Normalization, STFT	PyTorch	1D-CNN	--	Softmax	--
				2D-CNN			
[71]	TUH	Feature Extraction	TensorFlow	2D-CNN	10	Softmax	74.00
[72]	Clinical	Filtering, EMD, DWT, Fourier	Octave	2D-CNN	4	Sigmoid	99.50
	Bern Barcelona		Keras			Softmax	
[73]	Bern Barcelona	Normalization, STFT	TensorFlow	2D-CNN	10	Softmax	91.80
[74]	Bonn	DWT	NA	2D-CNN	10	Softmax	100
[75]	Bonn	CWT	Keras	2D-CNN	10	Softmax	100
[76]	Bonn	Filtering	Matlab	2D-CNN	--	Softmax	99.60
							90.10
[77]	CHB-MIT	FFT, WPD	TensorFlow	2D-CNN	5	MV-TSK-FS	98.35
			Matlab	3D-CNN			
[78]	Clinical	Different Methods	Matlab	2D-CNN	10	Sigmoid	NA
						RF	
[79]	CHB-MIT	MAS	NA	2D-CNN	5	KELM	99.33
	Clinical						
[80]	Clinical	Filtering, Down-Sampling	TensorFlow	1D-CNN	4	Softmax	83.86
						SVM	
[60]	Clinical	Different Techniques	Caffe	FRCNN with 2D-CNN	5	SVM	95.19
			Keras				
				FRCNN with 2D-CNN-LSTM			
			Theano			Sigmoid	
[57]	Bern Barcelona	NA	Caffe	Pre-Train Methods	--	Softmax	100
[58]	UCI	Signal2Image	PyTorch	1D-CNN	--	DenseNet	85.30
[86]	Bonn	DA	TensorFlow	P-1D-CNN	10	Majority Voting	99.10
[87]	Bonn	Normalization	Matlab	1D-CNN	10	Softmax	86.67
[88]	CHB-MIT	Filtering, DA	NA	MPCNN	--	Softmax	NA
[89]	Clinical	Down-Sampling, Filtering	Keras	1D-FCNN	5	Softmax	NA
[91]	TUH	Normalization	Keras	1D-CNN	--	Softmax	79.34
[90]	Clinical	Filtering	Theano	1D-CNN	--	Binary LR	NA
			Lasagne				
[81]	CHB-MIT	DWT, Feature Extraction, Normalization	NA	1D-CNN	10	--	99.07
[92]	Bonn	DWT, Normalization	NA	1D-CNN	5	Sigmoid	97.27
[85]	Bonn	Normalization	NA	1D-TCNN	NA	NA	100
[82]	Bonn	EMD, MPF	NA	1D-CNN	10	Softmax	98.60
[93]	CHB-MIT	Windowing	NA	IndRNN	10	NA	87.00
[94]	Bern Barcelona	Filtering, Normalization	TensorFlow	1D-CNN	--	Softmax	91.80
							99.00
	Bonn						
[83]	CHB-MIT	Filtering	PyTorch	1D-PCM-CNN	5	Softmax	NA
	Clinical						
[95]	CHB-MIT	MIDS, WGAN	NA	1D-CNN	--	Softmax	84.00
[96]	Clinical	Down-Sampling, PSD, FFT	NA	1D-CNN	4	Sigmoid	86.29
[97]	CHB-MIT	Filtering	TensorFlow	1D-CNN	4	Softmax	NA
[99]	Bern Barcelona	Filtering, DA	NA	1D-CNN	10	NA	89.28
[100]	Bonn	Normalization	Keras	1D-CNN	10	Softmax	98.67
			TensorFlow				
[101]	Clinical	Filtering, Normalization, Segmentation, resampling strategies	NA	Deep ConvNet	10	Softmax	80.00
[84]	Clinical	Down-Sampling, Filtering, DA	Keras	CNN-BP	5	Sigmoid	NA
			TensorFlow				
			Matlab				
[98]	Clinical	Filtering, DWT	NA	1D-CNN	--	Sigmoid	NA
				LSTM		RF	
				GRU		SVM	
[105]	CHB-MIT	Filtering, Montage Mapping	Matlab	DRNN	--	MLP	NA
[110]	Bonn	Filtering	NA	LSTM	--	Softmax	100
[106]	Bonn	Filtering	Keras	LSTM	3	Softmax	100
			TensorFlow		5		
			Matlab		10		
[107]	Bonn	Windowing	Keras	LSTM	10	Sigmoid	91.25
[108]	Bonn	Filtering	Keras	LSTM	3	Softmax	100
			TensorFlow		5		
			Matlab		10		
[109]	Freiburg	Filtering, Normalization	NA	LSTM	5	Softmax	97.75
[102]	CHB-MIT	Windowing	NA	ADIndRNN	10	NA	88.70
	Bonn						
[103]	Bonn	Autocorrelation	Keras	GRU	--	LR	98.00
[111]	Bonn	DWT	Keras	RNN	--	LR	98.50
[112]	Freiburg	Segmentation, DA, Stockwell Transform	Matlab	Bi-LSTM	--	Softmax	98.91
			TensorFlow				
[104]	TUH	TCP	NA	ChronoNet	--	Softmax	90.60
[113]	Clinical	Windowing	NA	AE with EM-PCA	--	GA	93.92
[114]	Bonn	Filtering, HWPT, FD	Matlab	AE	--	Softmax	98.67
[120]	Clinical	Down-Sampling, Filtering, Normalization	TensorFlow	AE	--	Sigmoid	NA
[121]	CHB-MIT	STFT	NA	SSDA	--	Softmax	93.82
[115]	Bonn	Normalization	Matlab	DSAE	--	LR	100
[116]	TUH	Different Methods	Toolkits	SDA	--	LR	NA
			Theano				
[117]	Bonn	Filtering	NA	SAE	--	SVM	100
[122]	Bonn	Normalization	NA	SSAE	--	Softmax	100
[118]	CHB-MIT	Scalogram	Theano	Wave2Vec	--	Softmax	93.92
[136]	CHB-MIT	DA, STFT	PyTorch	CNN-AE	5	Softmax	94.37
[123]	Clinical	Filtering, CWT, Feature Extraction	NA	SAE	--	Softmax	86.50
[124]	Bonn	Taguchi Method	NA	SSAE	--	Softmax	100
[125]	Clinical	Dimension Reduction, ESD	NA	DeSAE	--	Softmax	100
[126]	Bonn	DWT	NA	SAE	--	Softmax	96.00
[119]	CHB-MIT	Different Methods	NA	mSSDA	--	Softmax	96.61
[127]	Clinical	PCA, I-ICA	Matlab	SSAE	--	Softmax	94.00
[128]	Bonn	Windowing	Matlab	SAE	--	Softmax	88.80
[129]	Clinical	DWT	Matlab	DBN	--	--	96.87
[130]	Clinical	Normalization, Feature Extraction	Theano	DBN	--	LR	NA
						SVM	
						KNN	
[133]	CHB-MIT	Image Based Representation	NA	2D-CNN-LSTM	--	--	--
[131]	Clinical	Filtering	TensorFlow	ST-GRU ConNets	--	--	77.30
[132]	CHB-MIT	STFT, 2D-Mapping	NA	3D-CNN with Bi GRU	--	--	99.40
	Clinical						
[134]	CHB-MIT	Visualization	NA	2D-CNN-LSTM	--	Softmax	99.00
[135]	Clinical ECoG	Filtering	NA	1D-CNN-LSTM	5	Sigmoid	89.73
[138]	CHB-MIT	Channel Selection	NA	CNN-AE	5	Different Methods	92.00
	Bonn				10		
[139]	Bonn	Windowing	NA	1D-CNN with Bi LSTM	--	Softmax	99.33
						Sigmoid	100
[140]	Clinical	Mapping	Theano	ASAE-CNN	--	LR	68.00
				AAE-CNN			
[137]	CHB-MIT	STFT	PyTorch	CNN-AE	5	Softmax	96.22
[141]	SCTIMST	Noise reduction with BM3D, Skull stripping, Segmentation,	Keras	2D-CNN	5	Sigmoid	NA
			TensorFlow				
[142]	Clinical MRI	Different Techniques	NA	2D-CNN	5	Softmax	NA
[143]	Clinical MRI	Filtering, ICA, BCG, GLM, MCS	NA	ResNet	--	Softmax	NA
						Triplet	
[144]	Clinical Datasets	Different Methods	NA	2D-CNN	--	SVM	NA
[145]	Clinical MRI	Scaling Down	NA	3D-CNN	5	Softmax	89.80
[146]	Clinical MRI	Connectivity Feature extraction	NA	2D-CNN	--	--	--
[147]	Kaggle	ROI, Normalization, AAL, CNNI, Down-sampling, NNI (3D images)	TensorFlow	2D-ResNet	--	Sigmoid	98.22
				2D-VGG			
	Clinical MRI			2D-Inception V3			
				3D-SVGG-C3D			
[148]	Clinical MRI	OSEM, DA	TensorFlow	DAC	--	Tanh	NA

## References

[1] Ghassemi N., Shoeibi A., Rouhani M., Hosseini-Nejad H. Epileptic seizures detection in EEG signals using TQWT and ensemble learning. Proceedings of the 2019 9th International Conference on Computer and Knowledge Engineering (ICCKE).

[2] Shoeibi A., Ghassemi N., Alizadehsani R., Rouhani M., Hosseini-Nejad H., Khosravi A., Panahiazar M., Nahavandi S. (2021). A comprehensive comparison of handcrafted features and convolutional autoencoders for epileptic seizures detection in EEG signals. Expert Syst. Appl..

[3] Bhattacharyya A., Pachori R.B., Upadhyay A., Acharya U.R. (2017). Tunable-Q wavelet transform based multiscale entropy measure for automated classification of epileptic EEG signals. Appl. Sci..

[4] Kulaseharan S., Aminpour A., Ebrahimi M., Widjaja E. (2019). Identifying lesions in paediatric epilepsy using morphometric and textural analysis of magnetic resonance images. Clin. NeuroImage.

[5] Zazzaro G., Cuomo S., Martone A., Montaquila R.V., Toraldo G., Pavone L. (2019). Eeg signal analysis for epileptic seizures detection by applying data mining techniques. Internet Things.

[6] van Klink N., Mooij A., Huiskamp G., Ferrier C., Braun K., Hillebrand A., Zijlmans M. (2019). Simultaneous MEG and EEG to detect ripples in people with focal epilepsy. Clin. Neurophysiol..

[7] Pianou N., Chatziioannou S. (2019). Imaging with PET/CT in Patients with Epilepsy. Epilepsy Surgery and Intrinsic Brain Tumor Surgery.

[8] Subasi A., Kevric J., Canbaz M.A. (2019). Epileptic seizure detection using hybrid machine learning methods. Neural Comput. Appl..

[9] Acharya U.R., Oh S.L., Hagiwara Y., Tan J.H., Adeli H., Subha D.P. (2018). Automated EEG-based screening of depression using deep convolutional neural network. Comput. Methods Programs Biomed..

[10] Lauretani F., Longobucco Y., Ravazzoni G., Gallini E., Salvi M., Maggio M. (2021). Imaging the Functional Neuroanatomy of Parkinson’s Disease: Clinical Applications and Future Directions. Int. J. Environ. Res. Public Health.

[11] Carbó-Carreté M., Cañete-Massé C., Figueroa-Jiménez M.D., Peró-Cebollero M., Guàrdia-Olmos J. (2020). Relationship between Quality of Life and the Complexity of Default Mode Network in Resting State Functional Magnetic Resonance Image in Down Syndrome. Int. J. Environ. Res. Public Health.

[12] Morales Chacón L.M., González González J., Ríos Castillo M., Berrillo Batista S., Batista García-Ramo K., Santos Santos A., Cordero Quintanal N., Zaldívar Bermúdez M., Garbey Fernández R., Estupiñan Díaz B. (2021). Surgical Outcome in Extratemporal Epilepsies Based on Multimodal Pre-Surgical Evaluation and Sequential Intraoperative Electrocorticography. Behav. Sci..

[13] Takagi S., Sakuma S., Morita I., Sugimoto E., Yamaguchi Y., Higuchi N., Inamoto K., Ariji Y., Ariji E., Murakami H. (2020). Application of Deep Learning in the Identification of Cerebral Hemodynamics Data Obtained from Functional Near-Infrared Spectroscopy: A Preliminary Study of Pre-and Post-Tooth Clenching Assessment. J. Clin. Med..

[14] Ronan L., Alhusaini S., Scanlon C., Doherty C.P., Delanty N., Fitzsimons M. (2012). Widespread cortical morphologic changes in juvenile myoclonic epilepsy: Evidence from structural MRI. Epilepsia.

[15] Sharma R., Pachori R.B. (2015). Classification of epileptic seizures in EEG signals based on phase space representation of intrinsic mode functions. Expert Syst. Appl..

[16] Sheoran M., Kumar S., Chawla S. (2015). Methods of denoising of electroencephalogram signal: A review. Int. J. Biomed. Eng. Technol..

[17] Romaine J., Martín M.P., Ortiz J.S., Crespo J.M. (2021). EEG—Single-Channel Envelope Synchronisation and Classification for Seizure Detection and Prediction. Brain Sci..

[18] Perez-Sanchez A.V., Perez-Ramirez C.A., Valtierra-Rodriguez M., Dominguez-Gonzalez A., Amezquita-Sanchez J.P. (2020). Wavelet Transform-Statistical Time Features-Based Methodology for Epileptic Seizure Prediction Using Electrocardiogram Signals. Mathematics.

[19] Raschka S., Mirjalili V. (2017). Python Machine Learning: Machine Learning and Deep Learning with Python. Scikit-Learn and TensorFlow.

[20] Bonaccorso G. (2017). Machine Learning Algorithms.

[21] Gulli A., Pal S. (2017). Deep Learning with KERAS.

[22] Tang X., Zhang X. (2020). Conditional adversarial domain adaptation neural network for motor imagery EEG decoding. Entropy.

[23] Alickovic E., Kevric J., Subasi A. (2018). Performance evaluation of empirical mode decomposition, discrete wavelet transform, and wavelet packed decomposition for automated epileptic seizure detection and prediction. Biomed. Signal Process. Control.

[24] Sharma M., Bhurane A.A., Acharya U.R. (2018). MMSFL-OWFB: A novel class of orthogonal wavelet filters for epileptic seizure detection. Knowl. Based Syst..

[25] Mohammadpoor M., Shoeibi A., Shojaee H. (2016). A hierarchical classification method for breast tumor detection. Iran. J. Med. Phys..

[26] Assi E.B., Nguyen D.K., Rihana S., Sawan M. (2017). Towards accurate prediction of epileptic seizures: A review. Biomed. Signal Process. Control..

[27] Khodatars M., Shoeibi A., Ghassemi N., Jafari M., Khadem A., Sadeghi D., Moridian P., Hussain S., Alizadehsani R., ZARE A. (2020). Deep Learning for Neuroimaging-based Diagnosis and Rehabilitation of Autism Spectrum Disorder: A Review. arXiv.

[28] Sadeghi D., Shoeibi A., Ghassemi N., Moridian P., Khadem A., Alizadehsani R., Teshnehlab M., Gorriz J.M., Nahavandi S. (2021). An Overview on Artificial Intelligence Techniques for Diagnosis of Schizophrenia Based on Magnetic Resonance Imaging Modalities: Methods, Challenges, and Future Works. arXiv.

[29] Craik A., He Y., Contreras-Vidal J.L. (2019). Deep learning for electroencephalogram (EEG) classification tasks: A review. J. Neural Eng..

[30] Ghassemi N., Shoeibi A., Khodatars M., Heras J., Rahimi A., Zare A., Pachori R.B., Gorriz J.M. (2021). Automatic Diagnosis of COVID-19 from CT Images using CycleGAN and Transfer Learning. arXiv.

[31] Sharifrazi D., Alizadehsani R., Hassannataj Joloudari J., Shamshirband S., Hussain S., Alizadeh Sani Z., Hasanzadeh F., Shoaibi A., Dehzangi A., Alinejad-Rokny H. (2020). CNN-KCL: Automatic Myocarditis Diagnosis using Convolutional Neural Network Combined with K-means Clustering. Preprints.

[32] Srivastava N., Salakhutdinov R. (2012). Multimodal Learning with Deep Boltzmann Machines. NIPS.

[33] Yu D., Deng L. (2011). Deep Learning and Its Applications to Signal and Information Processing Exploratory DSP. IEEE Signal Process. Mag..

[34] Ihle M., Feldwisch-Drentrup H., Teixeira C.A., Witon A., Schelter B., Timmer J., Schulze-Bonhage A. (2012). EPILEPSIAE–A European epilepsy database. Comput. Methods Programs Biomed..

[35] Shoeb A.H. (2009). Application of Machine Learning to Epileptic Seizure onset Detection and Treatment.

[36] Seizure Prediction Challenge. https://www.kaggle.com/c/seizure-prediction.

[37] Andrzejak R.G., Lehnertz K., Mormann F., Rieke C., David P., Elger C.E. (2001). Indications of nonlinear deterministic and finite-dimensional structures in time series of brain electrical activity: Dependence on recording region and brain state. Phys. Rev. E.

[38] Andrzejak R.G., Schindler K., Rummel C. (2012). Nonrandomness, nonlinear dependence, and nonstationarity of elec-troencephalographic recordings from epilepsy patients. Phys. Rev. E.

[39] Stevenson N.J., Tapani K., Lauronen L., Vanhatalo S. (2019). A dataset of neonatal EEG recordings with seizure annotations. Sci. Data.

[40] Sharma R., Sircar P., Pachori R.B. (2019). Computer-aided diagnosis of epilepsy using bispectrum of EEG signals. Application of Biomedical Engineering in Neuroscience.

[41] Goodfellow I., Bengio Y., Courville A., Bengio Y. (2016). Deep Learning.

[42] Faust O., Hagiwara Y., Hong T.J., Lih O.S., Acharya U.R. (2018). Deep learning for healthcare applications based on physiological signals: A review. Comput. Methods Programs Biomed..

[43] Yildirim O., Talo M., Ay B., Baloglu U.B., Aydin G., Acharya U.R. (2019). Automated detection of diabetic subject using pre-trained 2D-CNN models with frequency spectrum images extracted from heart rate signals. Comput. Biol. Med..

[44] Martis R.J., Acharya U.R., Lim C.M., Mandana K.M., Ray A.K., Chakraborty C. (2013). Application of higher order cumulant features for cardiac health diagnosis using ECG signals. Int. J. Neural Syst..

[45] Pham T.-H., Vicnesh J., Wei J.K.E., Oh S.L., Arunkumar N., Abdulhay E.W., Ciaccio E.J., Acharya U.R. (2020). Autism Spectrum Disorder Diagnostic System Using HOS Bispectrum with EEG Signals. Int. J. Environ. Res. Public Health.

[46] Alizadehsani R., Roshanzamir M., Hussain S., Khosravi A., Koohestani A., Zangooei M.H., Abdar M., Beykikhoshk A., Shoeibi A., Zare A. (2021). Handling of uncertainty in medical data using machine learning and probability theory techniques: A review of 30 years (1991–2020). Ann. Oper. Res..

[47] Alizadehsani R., Sharifrazi D., Izadi N.H., Joloudari J.H., Shoeibi A., Gorriz J.M., Hussain S., Arco J.E., Sani Z.A., Khozeimeh F. (2021). Uncertainty-Aware Semi-supervised Method using Large Unlabelled and Limited Labeled COVID-19 Data. arXiv.

[48] Shoeibi A., Khodatars M., Alizadehsani R., Ghassemi N., Jafari M., Moridian P., Khadem A., Sadehi D., Hussain S., Zare A. (2020). Automated detection and forecasting of covid-19 using deep learning techniques: A review. arXiv.

[49] Krizhevsky A., Sutskever I., Hinton G.E. (2012). Imagenet classification with deep convolutional neural networks. Commun. ACM.

[50] Avcu M.T., Zhang Z., Chan D.W.S. Seizure detection using least eeg channels by deep convolutional neural network. Proceedings of the ICASSP 2019-2019 IEEE International Conference on Acoustics, Speech and Signal Processing.

[51] Hossain M.S., Amin S.U., Alsulaiman M., Muhammad G. (2019). Applying Deep Learning for Epilepsy Seizure Detection and Brain Mapping Visualization. ACM Trans. Multim. Comput. Commun. Appl..

[52] Zuo R., Wei J., Li X., Li C., Zhao C., Ren Z., Liang Y., Geng X., Jiang C., Yang X. (2019). Automated Detection of High-Frequency Oscillations in Epilepsy Based on a Convolutional Neural Network. Front. Comput. Neurosci..

[53] Asif U., Roy S., Tang J., Harrer S. (2020). SeizureNet: Multi-Spectral Deep Feature Learning for Seizure Type Classification. Machine Learning in Clinical Neuroimaging and Radiogenomics in Neuro-Oncology.

[54] Covert I.C., Krishnan B., Najm I., Zhan J., Shore M., Hixson J., Po M.J. Temporal graph convolutional networks for automatic seizure detection. Proceedings of the Machine Learning for Healthcare Conference.

[55] Bouaziz B., Chaari L., Batatia H., Quintero-Rincón A. (2019). Epileptic seizure detection using a convolutional neural network. Digital Health Approach for Predictive, Preventive, Personalised and Participatory Medicine.

[56] Deng J., Dong W., Socher R., Li L.J., Li K., Li F.F. Imagenet: A Large-Scale Hierarchical Image Database. Proceedings of the 2009 IEEE Conference on Computer Vision and Pattern Recognition.

[57] Taqi A.M., Al-Azzo F., Mariofanna M., Al-Saadi J.M. Classification and discrimination of focal and non-focal EEG signals based on deep neural network. Proceedings of the 2017 International Conference on Current Research in Computer Science and Information Technology (ICCIT).

[58] Bizopoulos P., Lambrou G.I., Koutsouris D. Signal2image modules in deep neural networks for eeg classification. Proceedings of the 2019 41st Annual International Conference of the IEEE Engineering in Medicine and Biology Society (EMBC).

[59] Simonyan K., Zisserman A. (2014). Very deep convolutional networks for large-scale image recognition. arXiv.

[60] Ahmedt-Aristizabal D., Fookes C., Nguyen K., Denman S., Sridharan S., Dionisio S. (2018). Deep facial analysis: A new phase I epilepsy evaluation using computer vision. Epilepsy Behav..

[61] Emami A., Kunii N., Matsuo T., Shinozaki T., Kawai K., Takahashi H. (2019). Seizure detection by convolutional neural network-based analysis of scalp electroencephalography plot images. NeuroImage Clin..

[62] Szegedy C., Liu W., Jia Y., Sermanet P., Reed S., Anguelov D., Erhan D., Vanhoucke V., Rabinovich A. Going deeper with convolutions. Proceedings of the IEEE Conference on Computer Vision and Pattern Recognition.

[63] Ayoobi N., Sharifrazi D., Alizadehsani R., Shoeibi A., Gorriz J.M., Moosaei H., Khosravi H., Nahavandi S., Chofreh A.G., Goni F.A. (2021). Time Series Forecasting of New Cases and New Deaths Rate for COVID-19 using Deep Learning Methods. arXiv.

[64] Antoniades A., Spyrou L., Took C.C., Sanei S. Deep learning for epileptic intracranial EEG data. Proceedings of the 2016 IEEE 26th International Workshop on Machine Learning for Signal Processing (MLSP).

[65] Achilles F., Tombari F., Belagiannis V., Loesch A.M., Noachtar S., Navab N. (2016). Convolutional neural networks for real-time epileptic seizure detection. Comput. Methods Biomech. Biomed. Eng. Imaging Vis..

[66] Park C., Choi G., Kim J., Kim S., Kim T.J., Min K., Jung K.-Y., Chong J. Epileptic seizure detection for multi-channel EEG with deep convolutional neural network. Proceedings of the 2018 International Conference on Electronics, Information, and Communication (ICEIC).

[67] Tjepkema-Cloostermans M.C., de Carvalho R.C., van Putten M.J. (2018). Deep learning for detection of focal epileptiform discharges from scalp EEG recordings. Clin. Neurophysiol..

[68] Golmohammadi M., Ziyabari S., Shah V., de Diego S.L., Obeid I., Picone J. (2017). Deep architectures for automated seizure detection in scalp EEGs. arXiv.

[69] Roy S., Kiral-Kornek I., Harrer S. Deep learning enabled automatic abnormal EEG identification. Proceedings of the 2018 40th Annual International Conference of the IEEE Engineering in Medicine and Biology Society (EMBC).

[70] Nejedly P., Kremen V., Sladky V., Nasseri M., Guragain H., Klimes P., Cimbalnik J., Varatharajah Y., Brinkmann B.H., Worrell G.A. (2019). Deep-learning for seizure forecasting in canines with epilepsy. J. Neural Eng..

[71] Iešmantas T., Alzbutas R. (2020). Convolutional neural network for detection and classification of seizures in clinical data. Med. Biol. Eng. Comput..

[72] San-Segundo R., Gil-Martín M., D’Haro-Enríquez L.F., Pardo J.M. (2019). Classification of epileptic EEG recordings using signal transforms and convolutional neural networks. Comput. Biol. Med..

[73] Sui L., Zhao X., Zhao Q., Tanaka T., Cao J. (2019). Localization of Epileptic Foci by Using Convolutional Neural Network Based on iEEG. IFIP International Conference on Artificial Intelligence Applications and Innovations.

[74] Akut R. (2019). Wavelet based deep learning approach for epilepsy detection. Health Inf. Sci. Syst..

[75] Türk Ö., Özerdem M.S. (2019). Epilepsy Detection by Using Scalogram Based Convolutional Neural Network from EEG Signals. Brain Sci..

[76] Liu J., Woodson B. Deep learning classification for epilepsy detection using a single channel electroencephalography (EEG). Proceedings of the 2019 3rd International Conference on Deep Learning Technologies.

[77] Tian X., Deng Z., Ying W., Choi K.-S., Wu D., Qin B., Wang J., Shen H., Wang S. (2019). Deep Multi-View Feature Learning for EEG-Based Epileptic Seizure Detection. IEEE Trans. Neural Syst. Rehabil. Eng..

[78] Ansari A.H., Cherian P.J., Caicedo A., Naulaers G., De Vos M., Van Huffel S. (2019). Neonatal Seizure Detection Using Deep Convolutional Neural Networks. Int. J. Neural Syst..

[79] Cao J., Zhu J., Hu W., Kummert A. (2020). Epileptic Signal Classification with Deep EEG Features by Stacked CNNs. IEEE Trans. Cogn. Dev. Syst..

[80] Thomas J., Comoretto L., Jin J., Dauwels J., Cash S.S., Westover M.B. EEG CLassification Via Convolutional Neural Network-Based Interictal Epileptiform Event Detection. Proceedings of the 2018 40th Annual International Conference of the IEEE Engineering in Medicine and Biology Society (EMBC), Honolulu, HI, USA, 18–21 July 2018.

[81] Boonyakitanont P., Lek-uthai A., Chomtho K., Songsiri J. (2019). A Comparison of Deep Neural Networks for Seizure Detection in EEG Signals. bioRxiv.

[82] Daoud H.G., Abdelhameed A.M., Bayoumi M. (2018). Automatic epileptic seizure detection based on empirical mode decomposition and deep neural network. Proceedings of the 2018 IEEE 14th International Colloquium on Signal Processing & Its Applications (CSPA).

[83] Craley J., Johnson E., Venkataraman A. (2019). Integrating convolutional neural networks and probabilistic graphical modeling for epileptic seizure detection in multichannel EEG. International Conference on Information Processing in Medical Imaging.

[84] Jaoude M.A., Jing J., Sun H., Jacobs C.S., Pellerin K.R., Westover M.B., Cash S.S., Lam A.D. (2020). Detection of mesial temporal lobe epileptiform discharges on intracranial electrodes using deep learning. Clin. Neurophysiol..

[85] Zhang J., Wu H., Su W., Wang X., Yang M., Wu J. A New Approach for Classification of Epilepsy EEG Signals Based on Temporal Convolutional Neural Networks. Proceedings of the 2018 11th International Symposium on Computational Intelligence and Design (ISCID), Hangzhou, China, 8–9 December 2018.

[86] Ullah I., Hussain M., Qazi E.-U.-H., Aboalsamh H. (2018). An automated system for epilepsy detection using EEG brain signals based on deep learning approach. Expert Syst. Appl..

[87] Acharya U.R., Oh S.L., Hagiwara Y., Tan J.H., Adeli H. (2018). Deep convolutional neural network for the automated detection and diagnosis of seizure using EEG signals. Comput. Biol. Med..

[88] Page A., Shea C., Mohsenin T. Wearable seizure detection using convolutional neural networks with transfer learning. Proceedings of the 2016 IEEE International Symposium on Circuits and Systems (ISCAS).

[89] O’Shea A., Lightbody G., Boylan G., Temko A. Neonatal seizure detection using convolutional neural networks. Proceedings of the 2017 IEEE 27th International Workshop on Machine Learning for Signal Processing (MLSP).

[90] Johansen A.R., Jin J., Maszczyk T., Dauwels J., Cash S.S., Westover M.B. Epileptiform spike detection via convolutional neural networks. Proceedings of the 2016 IEEE International Conference on Acoustics, Speech and Signal Processing (ICASSP).

[91] Yıldırım Ö., Baloglu U.B., Acharya U.R. (2018). A deep convolutional neural network model for automated identification of abnormal EEG signals. Neural Comput. Appl..

[92] Chen X., Ji J., Ji T., Li P. Cost-Sensitive Deep Active Learning for Epileptic Seizure Detection. Proceedings of the 2018 ACM International Conference on Bioinformatics, Computational Biology and Health Informatics, Washington, DC, USA, 2–4 May 2018.

[93] Yao X., Cheng Q., Zhang G.Q. (2019). A novel independent RNN approach to classification of seizures against non-seizures. arXiv.

[94] Lu D., Triesch J. (2019). Residual deep convolutional neural network for eeg signal classification in epilepsy. arXiv.

[95] Wei Z., Zou J., Zhang J., Xu J. (2019). Automatic epileptic EEG detection using convolutional neural network with improvements in time-domain. Biomed. Signal Process. Control.

[96] Meisel C., Atrache R.E., Jackson M., Schubach S., Ufongene C., Loddenkemper T. (2019). Deep learning from wristband sensor data: Towards wearable, non-invasive seizure forecasting. arXiv.

[97] Yuvaraj R., Thomas J., Kluge T., Dauwels J. A deep Learning Scheme for Automatic Seizure Detection from Long-Term Scalp EEG. Proceedings of the 2018 52nd Asilomar Conference on Signals.

[98] Fukumori K., Nguyen H.T.T., Yoshida N., Tanaka T. Fully Data-driven Convolutional Filters with Deep Learning Models for Epileptic Spike Detection. Proceedings of the ICASSP 2019-2019 IEEE International Conference on Acoustics, Speech and Signal Processing (ICASSP).

[99] Zhao X., Solé-Casals J., Li B., Huang Z., Wang A., Cao J., Tanaka T., Zhao Q. Classification of Epileptic IEEG Signals by CNN and Data Augmentation. Proceedings of the ICASSP 2020-2020 IEEE International Conference on Acoustics, Speech and Signal Processing (ICASSP).

[100] Abiyev R., Arslan M., Idoko J.B., Sekeroglu B., Ilhan A. (2020). Identification of epileptic eeg signals using convolutional neural networks. Appl. Sci..

[101] Lin L.-C., Ouyang C.-S., Wu R.-C., Yang R.-C., Chiang C.-T. (2019). Alternative Diagnosis of Epilepsy in Children without Epileptiform Discharges Using Deep Convolutional Neural Networks. Int. J. Neural Syst..

[102] Yao X., Cheng Q., Zhang G.Q. (2019). Automated Classification of Seizures against Nonseizures: A Deep Learning Approach. arXiv.

[103] Talathi S.S. (2017). Deep Recurrent Neural Networks for seizure detection and early seizure detection systems. arXiv.

[104] Roy S., Kiral-Kornek I., Harrer S. (2019). ChronoNet: A deep recurrent neural network for abnormal EEG identification. Conference on Artificial Intelligence in Medicine in Europe.

[105] Vidyaratne L., Glandon A., Alam M., Iftekharuddin K.M. Deep recurrent neural network for seizure detection. Proceedings of the 2016 International Joint Conference on Neural Networks (IJCNN).

[106] Hussein R., Palangi H., Ward R., Wang Z.J. (2018). Epileptic seizure detection: A deep learning approach. arXiv.

[107] Ahmedt-Aristizabal D., Fookes C., Nguyen K., Sridharan S. Deep Classification of Epileptic Signals. Proceedings of the 2018 40th Annual International Conference of the IEEE Engineering in Medicine and Biology Society (EMBC).

[108] Hussein R., Palangi H., Ward R.K., Wang Z.J. (2019). Optimized deep neural network architecture for robust detection of epileptic seizures using EEG signals. Clin. Neurophysiol..

[109] Jaafar S.T., Mohammadi M. (2019). Epileptic Seizure Detection using Deep Learning Approach. UHD J. Sci. Technol..

[110] Hussein R., Palangi H., Wang Z.J., Ward R. Robust detection of epileptic seizures using deep neural networks. Proceedings of the 2018 IEEE International Conference on Acoustics, Speech and Signal Processing (ICASSP).

[111] Verma A., Janghel R.R. (2021). Epileptic Seizure Detection Using Deep Recurrent Neural Networks in EEG Signals. Advances in Biomedical Engineering and Technology.

[112] Geng M., Zhou W., Liu G., Li C., Zhang Y. (2020). Epileptic Seizure Detection Based on Stockwell Transform and Bidirectional Long Short-Term Memory. IEEE Trans. Neural Syst. Rehabil. Eng..

[113] Rajaguru H., Prabhakar S.K. (2018). Multilayer Autoencoders and EM-PCA with Genetic Algorithm for Epilepsy Classification from EEG. Proceedings of the 2018 Second International Conference on Electronics, Communication and Aerospace Technology (ICECA), Coimbatore, India, 29–31 March 2018.

[114] Sharathappriyaa V., Gautham S., Lavanya R. (2018). Auto-encoder Based Automated Epilepsy Diagnosis. Proceedings of the 2018 International Conference on Advances in Computing, Communications and Informatics (ICACCI), Bangalore, India, 19–22 September 2018.

[115] Qiu Y., Zhou W., Yu N., Du P. (2018). Denoising Sparse Autoencoder Based Ictal EEG Classification. IEEE Trans. Neural Syst. Rehabil. Eng..

[116] Golmohammadi M., Torbati A.H.H.N., De Diego S.L., Obeid I., Picone J. (2019). Automatic Analysis of EEGs Using Big Data and Hybrid Deep Learning Architectures. Front. Hum. Neurosci..

[117] Yan B., Wang Y., Li Y., Gong Y., Guan L., Yu S. An EEG signal classification method based on sparse auto-encoders and support vector machine. Proceedings of the 2016 IEEE/CIC International Conference on Communications in China (ICCC).

[118] Yuan Y., Xun G., Suo Q., Jia K., Zhang A. (2019). Wave2Vec: Deep representation learning for clinical temporal data. Neurocomputing.

[119] Yuan Y., Xun G., Ma F., Suo Q., Xue H., Jia K., Zhang A. (2018). A novel channel-aware attention framework for multi-channel EEG seizure detection via multi-view deep learning. Proceedings of the 2018 IEEE EMBS International Conference on Biomedical & Health Informatics, Las Vegas, NV, USA, 4–7 March 2018.

[120] Emami A., Kunii N., Matsuo T., Shinozaki T., Kawai K., Takahashi H. (2019). Autoencoding of long-term scalp electroencephalogram to detect epileptic seizure for diagnosis support system. Comput. Biol. Med..

[121] Yuan Y., Xun G., Jia K., Zhang A. A multi-view deep learning method for epileptic seizure detection using short-time fourier transform. Proceedings of the 8th ACM International Conference on Bioinformatics, Computational Biology and Health Informatics.

[122] Lin Q., Ye S.Q., Huang X.M., Li S.Y., Zhang M.Z., Xue Y., Chen W.S. (2016). Classification of epileptic EEG signals with stacked sparse autoencoder based on deep learning. International Conference on Intelligent Computing.

[123] Gasparini S., Campolo M., Ieracitano C., Mammone N., Ferlazzo E., Sueri C., Tripodi G.G., Aguglia U., Morabito F.C. (2018). Information Theoretic-Based Interpretation of a Deep Neural Network Approach in Diagnosing Psychogenic Non-Epileptic Seizures. Entropy.

[124] Karim A.M., Güzel M.S., Tolun M.R., Kaya H., Çelebi F.V. (2018). A new generalized deep learning framework combining sparse autoencoder and Taguchi method for novel data classification and processing. Math. Probl. Eng..

[125] Karim A.M., Güzel M.S., Tolun M.R., Kaya H., Çelebi F.V. (2019). A new framework using deep auto-encoder and energy spectral density for medical waveform data classification and processing. Biocybern. Biomed. Eng..

[126] Karim A.M., Karal Ö., Çelebi F.V. (2018). A new automatic epilepsy serious detection method by using deep learning based on discrete wavelet transform.

[127] Hosseini M.P., Soltanian-Zadeh H., Elisevich K., Pompili D. Cloud-based deep learning of big EEG data for epileptic seizure prediction. Proceedings of the 2016 IEEE Global Conference on Signal and Information Processing (GlobalSIP).

[128] Singh K., Malhotra J. Stacked autoencoders based deep learning approach for automatic epileptic seizure detection. Proceedings of the 2018 First International Conference on Secure Cyber Computing and Communication (ICSCCC).

[129] Le T.X., Le T.T., Dinh V.V., Tran Q.L., Nguyen L.T., Nguyen D.T. (2018). Deep learning for epileptic spike detection. VNU J. Sci. Comput. Sci. Commun. Eng..

[130] Turner J.T., Page A., Mohsenin T., Oates T. (2017). Deep belief networks used on high resolution multichannel electroencephalography data for seizure detection. arXiv.

[131] Fang Z., Leung H., Choy C.S. Spatial temporal GRU convnets for vision-based real time epileptic seizure detection. Proceedings of the 2018 IEEE 15th International Symposium on Biomedical Imaging (ISBI 2018).

[132] Choi G., Park C., Kim J., Cho K., Kim T.-J., Bae H., Min K., Jung K.-Y., Chong J. A Novel Multi-scale 3D CNN with Deep Neural Network for Epileptic Seizure Detection. Proceedings of the 2019 IEEE International Conference on Consumer Electronics (ICCE).

[133] Thodoroff P., Pineau J., Lim A. (2016). Learning robust features using deep learning for automatic seizure detection. Mach. Learn. Healthc. Conf..

[134] Liang W., Pei H., Cai Q., Wang Y. (2020). Scalp EEG epileptogenic zone recognition and localization based on long-term recurrent convolutional network. Neurocomputing.

[135] RaviPrakash H., Korostenskaja M., Castillo E.M., Lee K.H., Salinas C.M., Baumgartner J., Anwar S.M., Spampinato C., Bagci U. (2020). Deep Learning provides exceptional accuracy to ECoG-based Functional Language Mapping for epilepsy surgery. Front. Neurosci..

[136] Yuan Y., Xun G., Jia K., Zhang A. (2018). A Multi-View Deep Learning Framework for EEG Seizure Detection. IEEE J. Biomed. Health Inform..

[137] Yuan Y., Jia K. (2019). FusionAtt: Deep Fusional Attention Networks for Multi-Channel Biomedical Signals. Sensors.

[138] Wen T., Zhang Z. (2018). Deep Convolution Neural Network and Autoencoders-Based Unsupervised Feature Learning of EEG Signals. IEEE Access.

[139] Abdelhameed A.M., Daoud H.G., Bayoumi M. (2018). Epileptic Seizure Detection using Deep Convolutional Autoencoder. Proceedings of the 2018 IEEE International Workshop on Signal Processing Systems (SiPS), Los Alamitos, CA, USA, 21–24 October 2018.

[140] Antoniades A., Spyrou L., Martin-Lopez D., Valentin A., Alarcon G., Sanei S., Took C.C. (2018). Deep neural architectures for mapping scalp to intracranial EEG. Int. J. Neural Syst..

[141] Dev K.B., Jogi P.S., Niyas S., Vinayagamani S., Kesavadas C., Rajan J. (2019). Automatic detection and localization of Focal Cortical Dysplasia lesions in MRI using fully convolutional neural network. Biomed. Signal Process. Control..

[142] Gill R.S., Hong S.J., Fadaie F., Caldairou B., Bernhardt B.C., Barba C., Brandt A., Coelho V.C., d’Incerti L., Lenge M. Deep convolutional networks for automated detection of epileptogenic brain malformations. Proceedings of the International Conference on Medical Image Computing and Computer-Assisted Intervention.

[143] Hao Y., Khoo H.M., von Ellenrieder N., Zazubovits N., Gotman J. (2018). DeepIED: An epileptic discharge detector for EEG-fMRI based on deep learning. NeuroImage Clin..

[144] Hosseini M.P., Tran T.X., Pompili D., Elisevich K., Soltanian-Zadeh H. Deep learning with edge computing for localization of epileptogenicity using multimodal rs-fMRI and EEG big data. Proceedings of the 2017 IEEE international conference on autonomic computing (ICAC).

[145] Yan M., Liu L., Chen S., Pan Y. (2018). A deep learning method for prediction of benign epilepsy with centrotemporal spikes. International Symposium on Bioinformatics Research and Applications.

[146] Gleichgerrcht E., Munsell B., Bhatia S., Vandergrift W.A., Rorden C., McDonald C., Edwards J., Kuzniecy R., Bonilha L. (2018). Deep learning applied to whole-brain connectome to determine seizure control after epilepsy surgery. Epilepsia.

[147] Jiang H., Gao F., Duan X., Bai Z., Wang Z., Ma X., Chen Y.W. (2019). Transfer Learning and Fusion Model for Classification of Epileptic PET Images. Innovation in Medicine and Healthcare Systems, and Multimedia.

[148] Shiri I., Ghafarian P., Geramifar P., Leung K.H.-Y., Ghelichoghli M., Oveisi M., Rahmim A., Ay M.R. (2019). Direct attenuation correction of brain PET images using only emission data via a deep convolutional encoder-decoder (Deep-DAC). Eur. Radiol..

[149] Rosas-Romero R., Guevara E., Peng K., Nguyen D.K., Lesage F., Pouliot P., Lima-Saad W.E. (2019). Prediction of epileptic seizures with convolutional neural networks and functional near-infrared spectroscopy signals. Comput. Biol. Med..

[150] Kiral-Kornek I., Roy S., Nurse E., Mashford B., Karoly P., Carroll T., Payne D., Saha S., Baldassano S., O’Brien T. (2018). Epileptic Seizure Prediction Using Big Data and Deep Learning: Toward a Mobile System. EBioMedicine.

[151] Alizadehsani R., Khosravi A., Roshanzamir M., Abdar M., Sarrafzadegan N., Shafie D., Khozeimeh F., Shoeibi A., Nahavandi S., Panahiazar M. (2020). Coronary Artery Disease Detection Using Artificial Intelligence Techniques: A Survey of Trends, Geographical Differences and Diagnostic Features 1991–2020. Comput. Biol. Med..

[152] Khozeimeh F., Sharifrazi D., Izadi N.H., Joloudari J.H., Shoeibi A., Alizadehsani R., Gorriz J.M., Hussain S., Sani Z.A., Moosaei H. (2021). CNN AE: Convolution Neural Network combined with Autoencoder approach to detect survival chance of COVID 19 patients. arXiv.

[153] Ghassemi N., Shoeibi A., Rouhani M. (2020). Deep neural network with generative adversarial networks pre-training for brain tumor classification based on MR images. Biomed. Signal Process. Control.

[154] Ghassemi N., Mahami H., Darbandi M.T., Shoeibi A., Hussain S., Nasirzadeh F., Alizadehsani R., Nahabandi D., Khosravi A., Nahavandi S. (2020). Material Recognition for Automated Progress Monitoring using Deep Learning Methods. arXiv.

[155] Yildirim O., Baloglu U.B., Acharya U.R. (2019). A Deep Learning Model for Automated Sleep Stages Classification Using PSG Signals. Int. J. Environ. Res. Public Health.

[156] Kim S., Kim J., Chun H.-W. (2018). Wave2Vec: Vectorizing Electroencephalography Bio-Signal for Prediction of Brain Disease. Int. J. Environ. Res. Public Health.

[157] Sarić R., Jokić D., Beganović N., Pokvić L.G., Badnjević A. (2020). FPGA-based real-time epileptic seizure classification using Artificial Neural Network. Biomed. Signal Process. Control.

[158] Saidi A., Othman S.B., Kacem W., Saoud S.B. FPGA Implementation of EEG Signal Analysis System for the Detection of epileptic seizure. Proceedings of the 2018 International Conference on Advanced Systems and Electric Technologies (IC_ASET).

[159] Feng L., Li Z., Wang Y. (2017). VLSI Design of SVM-Based Seizure Detection System with On-Chip Learning Capability. IEEE Trans. Biomed. Circuits Syst..

[160] Craley J., Johnson E., Jouny C., Venkataraman A. (2021). Automated inter-patient seizure detection using multichannel Convolutional and Recurrent Neural Networks. Biomed. Signal Process. Control.

[161] Martínez-Rodrigo A., García-Martínez B., Huerta Álvaro, Alcaraz R. (2021). Detection of Negative Stress through Spectral Features of Electroencephalographic Recordings and a Convolutional Neural Network. Sensors.

[162] Moore J.L., Carvalho D.Z., Louis E.K.S., Bazil C. (2021). Sleep and epilepsy: A focused review of pathophysiology, clinical syndromes, co-morbidities and therapy. Neurotherapeutics.

[163] Shoeibi A., Khodatars M., Jafari M., Moridian P., Rezaei M., Alizadehsani R., Khozeimeh F., Gorriz J.M., Heras J., Acharya U.R. (2021). Applications of Deep Learning Techniques for Automated Multiple Sclerosis Detection Using Magnetic Resonance Imaging: A Review. arXiv.

[164] Rim B., Sung N.J., Min S., Hong M. (2020). Deep learning in physiological signal data: A survey. Sensors.

[165] LeCun Y. 1.1 deep learning hardware: Past present and future. Proceedings of the 2019 IEEE International Solid-State Circuits Conference-(ISSCC).

[166] Haensch W., Gokmen T., Puri R. (2018). The next generation of deep learning hardware: Analog computing. Proc. IEEE.

[167] Tzallas A.T., Tsipouras M.G., Tsalikakis D.G., Karvounis E.C., Astrakas L., Konitsiotis S., Tzaphlidou M. (2012). Automated epileptic seizure detection methods: A review study. Epilepsy-Histol. Electroencephalogr. Psychol. Asp..

[168] Paul Y. (2018). Various epileptic seizure detection techniques using biomedical signals: A review. Brain Inform..

[169] Siddiqui M.K., Morales-Menendez R., Huang X., Hussain N. (2020). A review of epileptic seizure detection using machine learning classifiers. Brain Inform..

[170] Boonyakitanont P., Lek-Uthai A., Chomtho K., Songsiri J. (2020). A review of feature extraction and performance evaluation in epileptic seizure detection using EEG. Biomed. Signal Process. Control.

[171] Chakrabarti S., Swetapadma A., Pattnaik P.K. (2019). A review on epileptic seizure detection and prediction using soft computing techniques. Smart Techniques for a Smarter Planet.

[172] Rajendran T., Sridhar K.P. (2020). An overview of EEG seizure detection units and identifying their complexity-A review. Curr. Signal Transduct. Ther..

[173] Rasheed K., Qayyum A., Qadir J., Sivathamboo S., Kwan P., Kuhlmann L., O’Brien T., Razi A. (2020). Machine learning for predicting epileptic seizures using eeg signals: A review. IEEE Rev. Biomed. Eng..

[174] Abbasi B., Goldenholz D.M. (2019). Machine learning applications in epilepsy. Epilepsia.

[175] Acharya U.R., Hagiwara Y., Adeli H. (2018). Automated seizure prediction. Epilepsy Behav..

